# The changes of intestinal flora and metabolites in atopic dermatitis mice

**DOI:** 10.3389/fmicb.2024.1462491

**Published:** 2024-12-16

**Authors:** Feifei Wang, Zuding Wang, Liping Qu

**Affiliations:** ^1^Yunnan Botanee Bio-Technology Group Co., Ltd., Kunming, China; ^2^Yunnan Characteristic Plant Extraction Laboratory, Yunnan Yunke Characteristic Plant Extraction Laboratory Co., Ltd., Kunming, China; ^3^Innovation Materials Research and Development Center, Botanee Research Institute, Shanghai Jiyan Biomedical Development Co., Ltd., Shanghai, China

**Keywords:** atopic dermatitis, gut microbiota, intestinal bacteria metabolisms, linoleic acid, *Ruminococcaceae*

## Abstract

**Introduction:**

Atopic dermatitis (AD) is an allergic disease caused by various factors that can affect an individual’s appearance and cause psychological stress. Therefore, it is necessary to investigate the underlying mechanisms and develop effective treatment strategies. The gut microbiota and bacterial metabolism play crucial roles in human diseases. However, their specific role in AD remains unclear.

**Methods:**

In this study, we established a mouse model of AD and found that 2,4-dinitrofluorobenzene disrupted the skin barrier in mice. The species composition of intestinal bacteria was then analyzed by fecal 16s rRNA sequencing. The metabolic level of mice was analyzed by untargeted and targeted metabolomics in stool.

**Results:**

The levels of filaggrin and aquaporin 3 proteins in the model mice and total superoxide dismutase, catalase and malondialdehyde levels were significantly altered. Additionally, inflammatory factors such as tumor necrosis factor-alpha showed a significant increase. Using 16S rRNA gene sequencing, we identified 270 bacterial species with altered abundances of *Ruminococcaceae* and *Bifidobacteriaceae*. The untargeted metabolomic analysis detected 1,299 metabolites. Targeted analysis of free fatty acids revealed 49 metabolites with notable increases in linoleic and linolenic acid levels. Fecal bacterial transplantation experiments have demonstrated that oxidative stress, inflammation, and skin barrier damage were alleviated after transplantation.

**Discussion:**

These findings suggested that the metabolite linoleic acid negatively correlated with *Ruminococcaceae* and *Bifidobacteriaceae* may influence AD development. Perturbations in the intestinal bacteria and flora contributed to the development of AD, and the mouse model could serve as a valuable tool for further investigation of therapeutic approaches for managing ADS.

## 1 Introduction

At Atopic dermatitis (AD) is a chronic skin disease characterized by recurrent and acute flares, with primary symptoms including intractable pruritus, cutaneous eczema, structural defects, and immune dysfunction (Zeng et al., 2020). AD affects 1–3% of adults and is more prevalent in children (1–7 years old), affecting 10–20% (Wu et al., 2011). Increasing evidence suggests that AD is a systemic disease that is associated with cardiovascular, autoimmune, neurological, psychiatric, and metabolic disorders (Mesjasz et al., 2023). Considering the significant impact of AD on patients, it is crucial to investigate its pathogenesis and explore effective treatment methods.

The pathogenesis of AD typically involves five factors: disruption of the epidermal barrier, dysbiosis of the skin microbiota, altered immune responses, and genetic and environmental factors (Sroka-Tomaszewska and Trzeciak, 2021). Immune responses and genetic factors contribute to AD pathogenesis, with studies indicating a link between the incidence of AD and exposure to acidic oxides, nitrogen dioxide, and particulate matter (Park et al., 2022). Chronic skin inflammation in AD can lead to dysbiosis of the skin microbiota and increase pathogenic strains (Santos-Marcos et al., 2019).

The intestinal microenvironment, which is influenced by the gut microbiota and its products, significantly affects immune function. Specific diseases can alter the composition of the intestinal flora, and the microbiota in the intestine can influence diseases through various axes, such as the enterohepatic, intestinal-brain, intestinal-heart, and intestinal-skin axis (Zhou et al., 2020; Góralczyk-Bińkowska et al., 2022; Pabst et al., 2023; Matsiras et al., 2023; Qiao et al., 2024). Metabolites derived from the gut microbiota, particularly from fatty acid and tryptophan metabolism, play crucial roles at the cellular and systemic levels by interacting with the immune system and skin cell receptors (Stec et al., 2023). Several probiotics, including nitrobacteria, lactic acid bacteria, and bifidobacterial, are beneficial to the skin (Gao et al., 2023). *Staphylococcus aureus* has been shown to colonize significantly more skin surfaces in AD patients (Geoghegan et al., 2018). However, there is still insufficient data to characterize the pathogenesis and therapeutic approach of AD through gut microorganisms. This is particularly true for in-depth studies of flora-metabolite interactions, as well as metabolite-host interactions.

This study used mice to establish the AD model, aiming to investigate the changes of intestinal microenvironment (flora and metabolites) occurring in AD mice by 16 s rRNA gene sequencing and metabolomics technology. Then, the relationship between bacteria-metabolite interactions and ameliorative or pathogenic effects on AD was explore. It provided a basis for the in-depth study of AD pathogenesis and the development of therapeutic drug targets.

## 2 Materials and methods

### 2.1 Reagents and chemicals

The standard feed was obtained from Liaoning Changsheng Biotechnology Co., Ltd. (Shenyang, China). 2,4-Dinitrofluorobenzene (DNFB) was obtained from Shanghai Macklin Biochemical Technology Co., Ltd. (Shanghai, China). The bicinchoninic acid protein assay kit was obtained from Seven Biotech (Beijing, China). Absolute ethanol, methanol, chloroform, and isopropanol were obtained from Tianjin Kemiou Chemical Reagent Co. Ltd. (Tianjin, China). The primers used for RT-PCR were synthesized by Gene Pharma Company (Suzhou, China). Catalase (CAT), total superoxide dismutase (T-SOD), malondialdehyde (MDA), hydroxyproline (HYP) and hyaluronic acid (HA) detection kits were obtained from the Nanjing Jiancheng Institute of Biotechnology (Nanjing, China). Enzyme-linked immunoassays for collagen type I (COL-1) and ceramide (CER) detection kits were obtained from Jiangsu Meibiao Biotechnology Co., Ltd. (Nanjing, China). Horseradish peroxidase-conjugated goat anti-rabbit antibody was obtained from Proteintech Group, Inc. (Wuhan, China). The aquaporin 3 (AQP3) antibody was obtained from Affinity Biosciences (Jiangsu, China). Anti-filaggrin (FLG) antibody was purchased from GeneTex (USA).

### 2.2 Animals and ethical approval

Male C57BL/6J mice weighing 18–22 g (8 weeks old) were procured from Chang-Sheng Biotechnology Co., Ltd. (Shenyang, China). All experimental procedures adhered strictly to the PR China Legislation Regarding the Use and Care of Laboratory Animals, and all animal tests were approved by the Animal Care and Use Committee of Dalian Medical University (SYXK (Liao) 2018-0007). The mice were housed at a temperature of 22 ± 3°C with a humidity level of 55 ± 10%. They were allowed free movement throughout the experiment and maintained on a 12-h light–dark cycle to ensure access to adequate feed, fresh drinking water, and a clean environment.

### 2.3 DNFB-induced AD in mice

The mice were randomly divided into Control and Model groups with eight mice in each group. DNFB was dissolved in the solvent of acetone: olive oil = 4: 1. The back of the neck of the mice was first shaved over an area of approximately 2 × 2 cm. On days 1, 3, 7, 11, 16, 21, 28, and 30, the mice in Model group were treated by 50 μL of 0.5% DNFB solution to the shaved area using a pipette gun, which was applied evenly with the outer wall of the sterile gun tip, and the animals in Control group was coated with the same volume of blank solvent (Liang et al., 2023). Three days before the mice were executed, fecal samples were collected continuously. Fecal sample collection was performed in an ultra-clean bench. This was done by grasping the mice and gently massaging the abdomen to stimulate defecation, which was collected into sterile cryopreservation tubes and then snap-frozen in liquid nitrogen for 5 min before transferring them to a −80°C freezer. For serum samples, 1.5 mL sterile centrifuge tubes were used to collect venous blood from the eyes of mice, which was left to stand for half an hour, then centrifuged at 3500 r/min for 5 min, and the supernatant was aspirated into new 1.5 mL sterile centrifuge tubes, which were dispensed and transferred to a −80°C refrigerator. Skin tissue samples were collected from the drugged area using sterilized surgical instruments, placed in 2 mL sterile centrifuge tubes, and transferred to a −80°C refrigerator.

### 2.4 Measurement of biochemical indicators

The levels of T-SOD (superoxide dismutase assay kit, JianCheng, Nanjing, China), MDA (malondialdehyde assay kit, JianCheng, Nanjing, China), CAT (CATalase assay kit, JianCheng, Nanjing, China) in serum and the levels of HYP (hydroxyproline assay kit), COL-1 (human collagen type I ELISA commercial kit, Meibiao, Nanjing, China), CE (mouse ceramide ELISA commercial kit, Meibiao, Nanjing, China), and HA (hyaluronic acid assay kit, JianCheng, Nanjing, China) in the skin tissue of mice were detected using commercial kits according to the manufacturer’s instructions.

### 2.5 Histopathological testing

Skin tissues were fixed in 10% formalin and stained with hematoxylin and eosin, Masson’s trichrome, and toluidine blue. Stained sections were observed under a light microscope (Nikon Eclipse E100, Tokyo, Japan) at 200× magnification.

### 2.6 Immunofluorescence assay

Paraffin sections of skin tissues were deparaffinized, treated with ethanol and phosphate-buffered saline (PBS), boiled in an antigen repair solution, washed with PBS, incubated in a wet box for 1 h, and then incubated with antibodies at 4°C for 12 h. Samples were rinsed with PBS, incubated with fluorescein-labeled secondary antibodies, stained with DAPI, and imaged using an upright fluorescence microscope (Nikon Eclipse C1, Japan).

### 2.7 RT-PCR assay

RNA samples were extracted from mouse skin, and cDNA was synthesized from total RNA using the EasyScript All-in-One First Strand cDNA Synthesis SuperMix kit. mRNA levels were quantified using the Bio-Rad CFX Maestro system with glyceraldehyde-3-phosphate dehydrogenase for normalization, and the 2^−△△Ct^ method was used for analysis.

### 2.8 Western blot assay

Skin tissue lysates were prepared, and protein concentrations were determined using a bicinchoninic acid kit. Samples were mixed with 6 × loading buffer, heated at 95°C for 5 min, separated using sodium dodecyl sulfate-polyacrylamide gel electrophoresis gel, transferred onto polyvinylidene fluoride membranes, blocked with blocking solution for 2 h, incubated with primary antibodies overnight at 4°C, incubated with secondary antibodies for 2 h at 4°C, and visualized using an enhanced chemiluminescence reagent on the ChemiDoc™ XRS Imaging System (Bio-Rad Laboratories, USA).

### 2.9 16S rRNA gene sequencing of feces samples

The integrity of the extracted genomic DNA and the concentration and purity of fecal samples were assessed (MagAtrract PowerSoil Pro DNA kit, Qiagen, Germany). The purity of DNA was assessed by determining the ratio of 260 nm/280 nm (≈1.8) and the ratio of 260 nm/230 nm (NanoDrop One, Thermo Fisher Scientific, Waltham, USA). The extracted DNA served as the template for PCR amplification of the V3-V4 variable region of the 16S rRNA gene, utilizing the upstream primer 338F and the downstream primer 806R, which carried the barcode sequence (Liang et al., 2023). Subsequently, purified PCR products were prepared using the NEXTFLEX Rapid DNA-Seq Kit and sequenced on an Illumina PE300 platform.

Noise reduction of optimized sequences after QC splicing was performed using the DADA2 plugin in the Qiime2 process based on default parameters. Based on the Sliva 16S rRNA gene database (v 138), the ASVs were analyzed for species taxonomy using the Naive bayes (or Vsearch, or Blast) classifier in Qiime2. All data analyses were performed on the Majorbio Cloud platform.^1^

### 2.10 Untargeted metabolism assay of feces samples in mice by GC-TOF/MS

Fecal samples were extracted before GC-TOF/MS analysis (Tan et al., 2022). The supernatant was dried using nitrogen and added methoxypyridine hydrochloride solution at 37°C for 90 min. Subsequently, the N,O-bis(trimethylsilyl)trifluoroacetamide derivatization reagent containing 1% trimethylchlorosilane was added and reacted at 70°C for 60 min. The analysis was conducted using an Agilent 8890 B gas chromatography system coupled with an Agilent 5977 B mass-selective detector. The sample was separated using a DB-5MS capillary column (40 m × 0.25 mm × 0.25 μm, Agilent). After onboarding, the raw GC/MS data were processed by the MassHunter workstation Quantitative Analysis (v10.0.707.0) software to produce a three-dimensional CSV-formatted data matrix containing the sample information, metabolite names, and mass spectral response intensities. Internal standard peaks as well as any known false positives (including noise, column loss, and derivatization reagent peaks) were removed from the data matrix, de-redundant, and peaks were merged. The metabolites were also identified by searching public databases such as NIST (2017), Fiehn (2013), MS-DIAL (2021). All data analyses were performed on the Majorbio Cloud platform (see text footnote 1).

### 2.11 Untargeted metabolism assay of feces samples in mice by UPLC-MS/MS

The samples were obtained from mouse feces for analysis (Liu et al., 2023), which was conducted using a Thermo UHPLC-Q Exactive HF-X system (Thermo Fisher Scientific, USA) equipped with an ACQUITY HSS T3 column (100 mm × 2.1 mm i.d., 1.8 μm, Waters, USA). The mobile phase consisted of 0.1% formic acid in water: acetonitrile (95:5, v/v) as mobile phase A and 0.1% formic acid in acetonitrile: isopropanol: water (47.5:47.5:5, v/v/v) as mobile phase B. During positive detection, the gradient of mobile phase B was increased from 0 to 20% (0–3 min), 20 to 35% (3–4.5 min), and 35 to 100% (4.5–5 min), and maintained at 100% (5–6.3 min). Conversely, for negative detection, the gradient of mobile phase B was increased from 0 to 5% (0–1.5 min), 5 to 10% (1.5–2 min), 10 to 30% (2–4.5 min), and 30 to 100% (4.5–5 min). The flow rate was set at 0.4 mL/min, and the column temperature was maintained at 40°C.

After on-boarding, the UPLC-MS/MS raw data were imported into the metabolomics processing software Progenesis QI (Waters Corporation, Milford, USA) for baseline filtering, peak identification, integration, retention time correction, peak alignment, and finally a data matrix of retention time, mass-to-charge ratio, and peak intensity was obtained. At the same time, the MS mass spectrometry information was matched with the metabolic public databases, HMDB^2^ and Metlin^3^, as well as Majorbio’s own libraries, to obtain the metabolite information. All data analyses were performed on the Majorbio Cloud platform (see text footnote 1).

### 2.12 Targeted metabolomics profiling of feces samples using GC-TOF/MS assay

Targeted metabolomics was performed using GC-TOF/MS for absolute quantification of a total of 32 free fatty acids (Yi et al., 2022). The free fatty acids were converted to fatty acid methyl esters after derivatization to improve their volatility and thermal stability, which could be detected by gas chromatographic analysis. The samples were extracted using a mixture of isopropanol and n-hexane (2:3, v/v containing 0.2 mg/L internal standard). They were then homogenized at 40 Hz for 4 min, sonicated in ice water for 5 min, and centrifuged at 12,000 rpm/4°C for 15 min. The resulting supernatant was collected and dried under a nitrogen atmosphere. Subsequently, a solution of methanol and trimethylsilyl diazomethane (1:2, v/v) was added to the dried sample, which was then dried again. The sample was then redissolved in n-hexane and centrifuged at 12,000 rpm for 1 min. Finally, the supernatant underwent GC–MS analysis using a 7890B GC System and a 5977B Mass Spectrometer (both from Agilent, USA), with separation achieved on a DB-FastFAME capillary column (90 m × 250 μm × 0.25 μm).

### 2.13 Fecal microbiome transplantation in mice

Male C57BL/6J mice weighing 18–22 g (8 weeks old) were procured from Chang- Sheng Biotechnology Co., Ltd. (Shenyang, China). Fecal bacterial transplantation experiments were performed by dividing mice into donor and recipient groups. Recipient mice were treated with 200 μL of triple antibiotic consisting of vancomycin (0.5 mg/mL), ampicillin (1 mg/mL) and metronidazole (1 mg/mL) for three consecutive days before the experiment. Donor mice were divided into Control (*n* = 5) and Model (*n* = 5) groups. All mice in the recipient group were modeled with 0.5% DNFB to establish AD and randomly divided into Control + DNFB (*n* = 5) and Model + DNFB (*n* = 5) groups. Fresh mouse feces from Control and Model groups were then transplanted into the recipient animals every 2 days. Each time, 200 mg of feces from donor group was taken, dissolved into 2 mL of sterile saline and centrifuged at 3500 r/min for 5 min to produce the supernatant. Each time, 100 μL of supernatant was given to the recipient mice by gavage. At the end of the test, the serum and the kin tissues were collected and stored with the at −80°C.

### 2.14 Correlation analysis

Metabolites identified using non-targeted metabolomics and differential bacteria identified using 16S rRNA gene sequencing were analyzed to determine the correlations. Data analysis was performed using the Majorbio Cloud Platform (see text footnote 1).

### 2.15 Statistical analysis

Images were processed using Image J software (version 1.53). Statistical analyses were conducted using GraphPad Prism software (version 5.0; San Diego, CA, USA). Unpaired t-tests were used to compare two groups. Data are presented as mean ± standard deviation, and *p*-values less than 0.05 or 0.01 were considered statistically significant.

## 3 Results

### 3.1 AD mice showed elevated levels of inflammation, oxidative stress, and disrupted skin barrier

**Figure 1:**
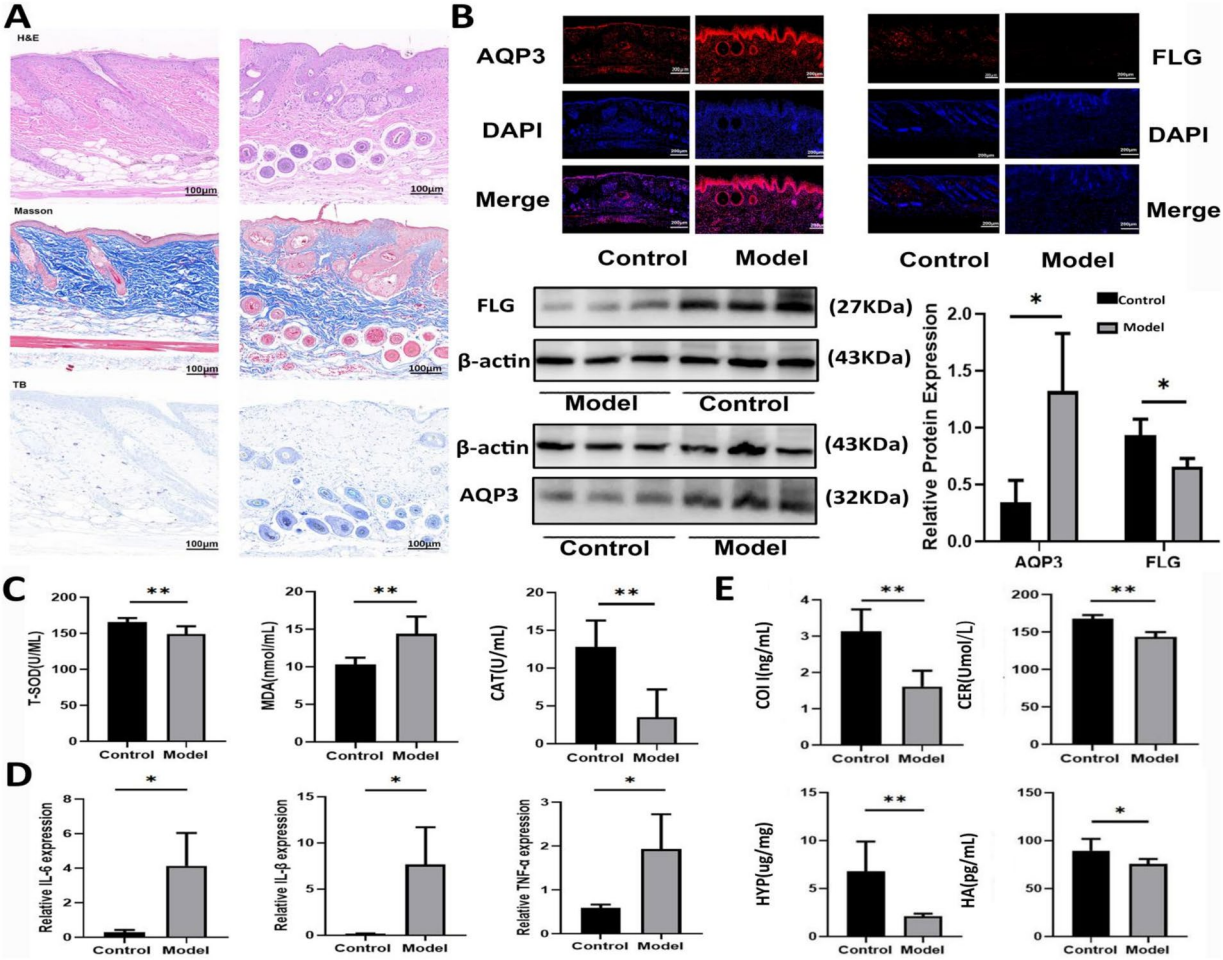
Establishment of an AD model in mice. **(A)** DNFB induced skin injury based on HE, Masson, and toluidine blue staining. **(B)** Expression levels of AQP3 and FLG in mice induced by DNFB based on western blot and immunofluorescence tests. Data are presented as the mean ± SD (*n* = 3). **(C)** The levels of T-SOD, MDA and CAT in mice induced by DNFB. Data are presented as the mean ± SD (*n* = 6). **(D)** The mRNA levels of IL-6, IL-1β and TNF-*α* in mice induced by DNFB. Data are presented as the mean ± SD (*n* = 3). **(E)** The levels of COL I, HA, CER and HYP in mice induced by DNFB. Data are presented as the mean ± SD (*n* = 6). ^*^p < 0.05, ^**^p < 0.01 compared with Model group.

As shown in Figure 1A, the skin of mice in Model group exhibited bloating and thickening of the epidermal and dermal layers. The dermal-epidermal junction appeared flattened, with a few twisted, broken, and disordered collagen fiber bundles in the dermis. Additionally, there was an increase in the number of mast cells, indicating damage to the skin barrier. As shown in Figure 1B, the level of FLG in the skin tissue of Model mice was significantly decreased, whereas the AQP3 level was markedly elevated compared with that in Control group. As shown in Figure 1C, compared with Control group, the levels of T-SOD and CAT significantly decreased, and the MDA level markedly increased in Model mice. In Figure 1D, the levels of inflammatory factors, including interleukin (IL)-6, IL-1β, and tumor necrosis factor-alpha, were elevated in model mice, while the levels of HYP, HA, COI-1, and CER in the skin tissue were significantly decreased (Figure 1E).

### 3.2 Changes of the intestinal flora in AD mice

**Figure 2:**
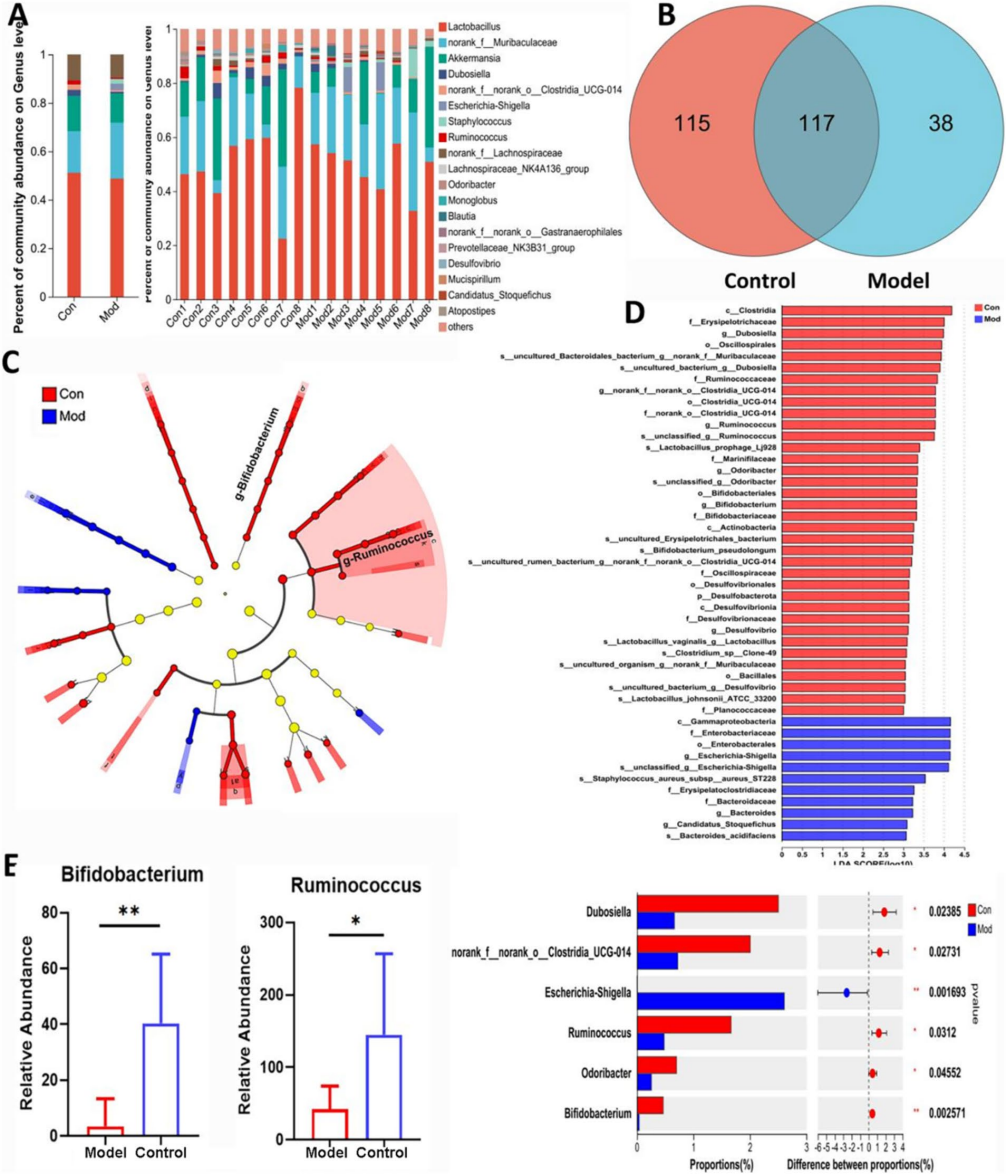
Gut microbiota composition in AD mice. **(A)** The microbial composition and relative abundance in Control and Model groups based on Community Barplot analysis. **(B)** Venn diagram illustrated the common and differential 270 microbiota among Control and Model groups. **(C)** Lefse assay showed the bacteria with greatly changes at different levels. **(D)** The differential flora marked in red or blue at the different levels in Control and Model groups, as well as the more differentiated bacteria at the genus level. **(E)** The bacteria involving the dominant species in Control and Model groups. Data are presented as the mean ± SD (*n* = 8).^*^p < 0.05, ^**^p < 0.01 compared with Model group.

The results of 16 s rRNA gene sequencing are presented in Figure 2. The data in Figure 2A show information on the top 20 genera in terms of abundance at the genus level. *Lactobacillus* was the most abundant genus in both Control and Model groups. Figure 2B shows the Veen plot, where a total of 270 microorganisms were detected, and 117 were co-occurring in both Control and Model groups, and Figures 2C,D shows the Linear discriminant analysis Effect Size (LEfSe) analysis, which showed a greater predominance of *Bifidobacterium* and *Ruminococcus* in Control group compared to Model group. Figure 2E showed the statistical analysis of genus differences, with significantly higher abundance of *Bifidobacterium* and *Ruminococcus* in Control group compared to Model group.

### 3.3 DNFB affected fecal metabolite levels in AD mice

**Figure 3:**
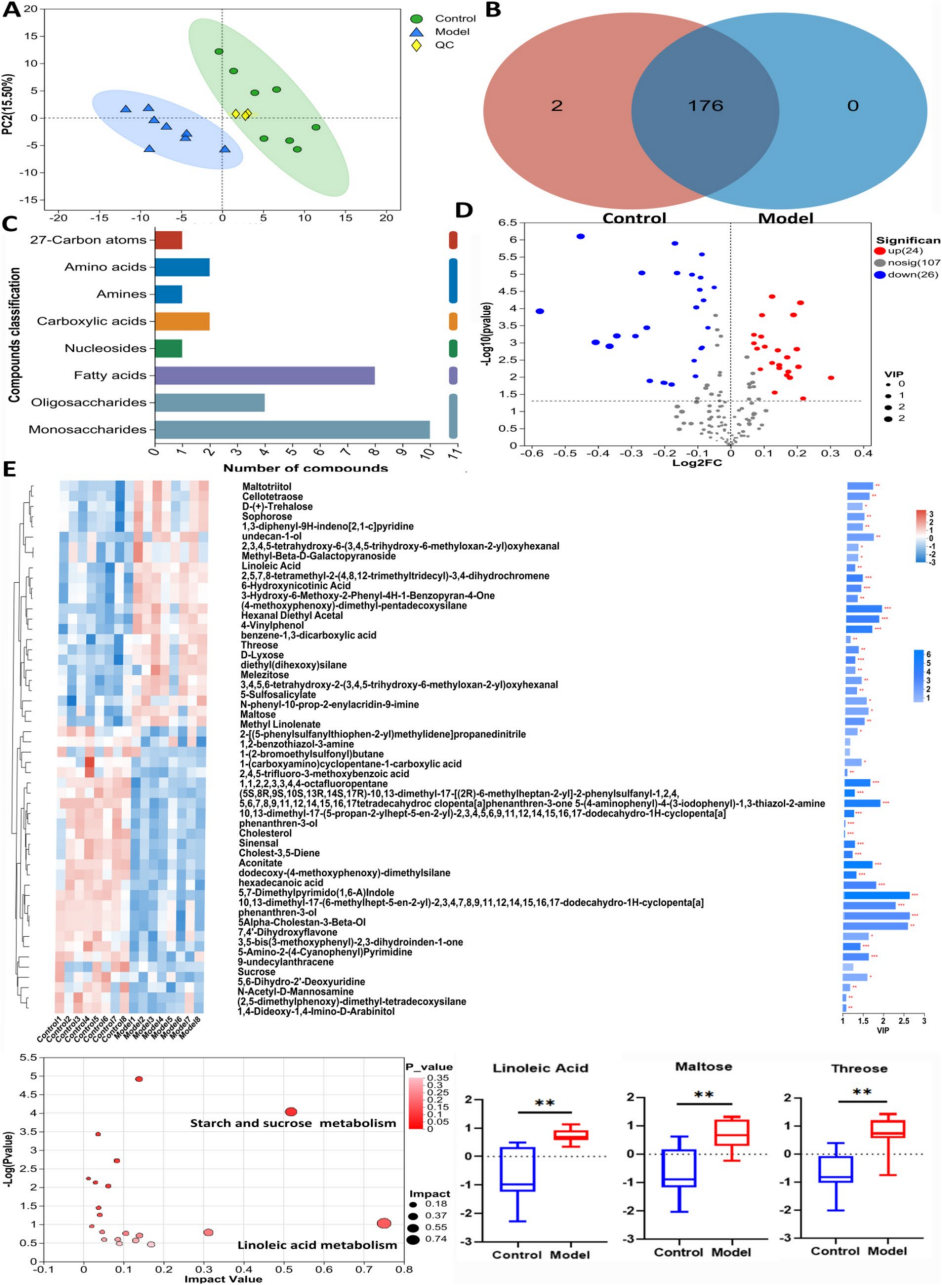
The untargeted metabolism of the feces in mice using GC/MS test. **(A)** The PCA plot showed a good separation of the samples in the two groups. **(B)** Venn diagram illustrated the common and differential 178 metabolites between the groups. **(C)** KEGG analysis showed the important pathways involved in the differential metabolites. **(D)** Volcano plot of the 50 differential metabolites between the groups. **(E)** Heatmap diagram showed the change trends of the differential metabolites, and the relative levels of the important metabolites and some pathways in Control and Model groups. Data are presented as the mean ± S. D (*n* = 8). ^**^p < 0.01 compared with Model group.

GC-TOF/MS detection identified 178 compounds in mouse feces. The differential metabolites are listed in Table 1. As shown in Figure 3A, principal component analysis (PCA) revealed significant differences in metabolite profiles between Control and Model groups. The Venn diagram in Figure 3B illustrates that 176 metabolites were common in both Control and Model groups. Figure 3C shows that fatty acid analogs and monosaccharides were the most abundant among the detected metabolites. The volcano map (Figure 3D) highlights 24 upregulated and 26 downregulated compounds compared with Control group (Log_2_FC < 1.5, *p* < 0.05). Figure 3E shows the cluster analysis, statistical analysis of variance, and KEGG pathway enrichment analysis of the differential metabolites. The metabolite levels of linoleic acid, maltose and threonine were significantly increased in the model group compared with the control group. In KEGG pathway enrichment, the differential metabolites in the control and model groups were mainly enriched in the pathways of linoleic acid metabolism, and metabolism of starch and sucrose.

**Table 1:** The metabolites in untargeted metabolism using GC/MS assay.

Metabolite	*p*-value	Fold change ^a^
Hexanal diethyl acetal	0.0001558	1.1405
Maltose	0.001671	1.1037
Threose	0.00131	1.074
Linoleic acid	0.001043	1.0499
(4-Methoxyphenoxy)-dimethyl-pentadecoxysilane	0.00006887	1.1573
Aconitate	0.000001284	0.8901
4-Vinylphenol	0.00004539	1.0906
Maltotriitol	0.001549	1.1485
Cellotetraose	0.002681	1.1256
D-(+)-Trehalose	0.04235	1.1637
Sophorose	0.004513	1.1069
1,3-Diphenyl-9H-indeno[2,1-c]pyridine	0.007016	1.1283
2,4,5-Trifluoro-3-methoxybenzoic acid	0.00000937	0.894
1,1,2,2,3,3,4,4-Octafluoropentane	0.00000268	0.9417
5,6-Dihydro-2′-deoxyuridine	0.01658	0.884
N-Acetyl-D-mannosamine	0.009544	0.9292
(2,5-dimethylphenoxy)-dimethyl-tetradecoxysilane	0.003356	0.9266
Undecan-1-ol	0.005061	1.1524
2,3,4,5-Tetrahydroxy-6-(3,4,5-trihydroxy-6-methyloxan-2-yl)oxyhexanal	0.02848	1.0966
Methyl-beta-D-galactopyranoside	0.02845	1.0964
2,5,7,8-tetramethyl-2-(4,8,12-trimethyltridecyl)-3,4-dihydrochromene	0.0001583	1.0679
6-Hydroxynicotinic acid	0.0006661	1.0659
3-Hydroxy-6-methoxy-2-phenyl-4H-1-benzopyran-4-one	0.001494	1.0568
Benzene-1,3-dicarboxylic acid	0.005963	1.0641
D-Lyxose	0.0005913	1.05
Diethyl(dihexoxy)silane	0.008925	1.1247
Melezitose	0.005494	1.1071
3,4,5,6-Tetrahydroxy-2-(3,4,5-trihydroxy-6-methyloxan-2-yl)oxyhexanal	0.003902	1.0906
5-Sulfosalicylate	0.01058	1.2336
N-phenyl-10-prop-2-enylacridin-9-Imine	0.0105	1.1319
9-Undecylanthracene	0.0006455	0.8196
1,4-Dideoxy-1,4-imino-d-arabinitol	0.001379	0.9423
Sinensal	0.0000289	0.9376
Cholest-3,5-diene	0.0000585	0.945
Dodecoxy-(4-methoxyphenoxy)-dimethylsilane	0.0000105	0.9225
hexadecanoic acid	0.0003861	0.8392
(5S,8R,9S,10S,13R,14S,17R)-10,13-dimethyl-17-[(2R)-6-methylheptan-2-yl]-2-phenylsulfanyl-1,2,4,5,6,7,8,9,11,12,14,15,16,17-tetradecahydrocyclopenta[a]phenanthren-3-one	0.00000931	0.8306
5-(4-Aminophenyl)-4-(3-iodophenyl)-1,3-thiazol-2-amine	0.0000127	0.9392
5,7-Dimethylpyrimido(1,6-a)indole	0.000000801	0.7309
10,13-Dimethyl-17-(6-methylhept-5-en-2-yl)-2,3,4,7,8,9,11,12,14,15,16,17-dodecahydro-1H-cyclopenta[a]phenanthren-3-ol	0.0006366	0.7883
5Alpha-cholestan-3-beta-ol	0.0009802	0.7542
7,4’-Dihydroxyflavone	0.001275	0.7765
5-Amino-2-(4-cyanophenyl)pyrimidine	0.000094	0.9307
10,13-Dimethyl-17-(5-propan-2-ylhept-5-en-2-yl)-2,3,4,5,6,9,11,12,14,15,16,17-dodecahydro-1H-cyclopenta[a]phenanthren-3-ol	0.0003687	0.9537
1-(2-Bromoethylsulfonyl)butane	0.01468	0.8691
1-(Carboxyamino)cyclopentane-1-carboxylic acid	0.001489	0.94
Methyl linolenate	0.0149	0.8713
3,5-Bis(3-methoxyphenyl)-2,3-dihydroinden-1-one	0.01299	0.8446
Oleyl amide	0.0001217	0.6712
Cholesterol	0.0000246	0.9662

a Average of model/average of control.

The metabolites in feces were determined using UPLC-MS/MS. In positive detection mode, 683 compounds were detected. In negative mode, 438 compounds were identified. Further details of other differential metabolites are listed in Tables 2, 3.

**Table 2:** The differential metabolites in untargeted metabolism under LC/MS test under positive detection.

Metabolite	*p*_value	Fold change^a^
Dihydroceramide	0.0000313	0.8671
Lys Lys Tyr Gly	0.00000768	1.3147
Thymidine	0.002491	1.1461
L-Serine	0.000104	0.879
N-Undecanoylglycine	0.0000736	0.6866
(−)-Stercobilin	0.002687	0.7945
3-Pyrimidin-2-yl-2-pyrimidin-2-ylmethyl-Propionic acid	0.000000426	2.7699
1-Radyl-2-acyl-sn-glycphocholinero-3-phose	0.0000192	1.5367
3b,12a-Dihydroxy-5a-cholanoic acid	0.0000122	0.8729
(Z)-Resveratrol 4′-glucoside	0.001392	1.2159
1-Acetoxy-2-hydroxy-16-heptadecyn-4-one	0.0000004585	1.1141
3-(1,2-Dihydroxybut-3-en-1-yl)-1H-isochromen-1-one	0.0000701	1.1302
5,7-Dihydroxy-2-(3-hydroxyphenyl)-4H-chromen-4-one	0.000773	1.2531
Wogonin	0.009406	1.1466
1b,3a,7b-Trihydroxy-5b-cholanoic acid	0.0000331	0.8957
Methyl 9,10-epoxy-12,15-octadecadienoate	0.0000000564	1.1993
Noralfentanil	0.001128	1.3219
Alanyl-glutamic acid	0.007024	1.1662
Asparaginyl-glutamic acid	0.001311	1.1603
Norepinephrine (noradrenaline)	0.000007838	1.1055
5-Hydroxymethyluracil	0.001585	1.1863
10-Hydroxymyristic acid methyl ester	0.001353	0.8927
Asp Pro Lys Leu	0.008003	1.3047
N-Acetyl-7-O-acetylneuraminic acid	0.005302	1.1881
Tryptophanol	0.0005714	0.7295
Trans-Piceid	0.002168	1.1946
Pelargonidin	0.0004757	1.265
Octadecanamide	0.0000143	1.3349
9,10,13-TriHOME	0.0000215	1.1151
Ile Val Leu Thr	0.007643	0.817
Penaresidin A	0.000001347	0.8842
DG(16:0/18:4(6Z,9Z,12Z,15Z)/0:0)	0.0000009399	1.3105
Jimenezin	0.000005596	1.1335
3-beta-Hydroxy-4-beta-methyl-5-alpha-cholest-7-ene-4-alpha-carboxylate	0.000488	0.894
3-Buten-2-one 1-(2,3,6-trimethyl phenyl)	0.00007061	0.8862
N-Acetyltyramine	0.0006793	0.8898
LysoPC(22:6(4Z,7Z,10Z,13Z,16Z,19Z))	0.005064	0.8883
5,8-Epoxy-5,8-dihydro-3-hydroxy-8′-apo-b,y-carotenal	0.002153	1.1332
3-O-Methylniveusin A	0.000003551	0.7155
(1E)-1-(3,4-dihydroxyphenyl)-7-(4-hydroxyphenyl)hept-1-ene-3,5-dione	0.0000044	1.4604
Dihydrodaidzein	0.006332	1.3421
Trandolapril-d5 diketopiperazine	0.0001495	1.2226
Liqcoumarin	0.009832	3.0888
Isoleucyl-Threonine	0.002471	0.736
Cassythine	0.0007759	1.3748
Lys Ala Ser Tyr	0.0003774	1.1577
Vanilloside	0.001919	1.1992
3,4,5-Trihydroxy-6-(2-methyl-3-oxo-1-phenylpropoxy)oxane-2-carboxylic acid	0.007912	1.3424
N-Acetylserotonin	0.000005031	0.8258
Tetradecanedioic acid	0.003833	0.8508
N-(4-aminobutyl)-3-(4-hydroxy-3-methoxyphenyl)propanimidic acid	0.0000007698	0.7586
Gerrardine	0.000002907	0.8736
Ser Tyr Val Val	0.004217	0.7984
1,2-Anhydridoniveusin	0.0002604	0.7643
Artabsinolide D	0.001243	1.2295
Hypoglycin B	0.003568	1.1492
PhistidinalIpemidic acid	0.00001845	1.4087
Histidinal	0.00002159	1.1312
Serinyl-Hydroxyproline	0.003301	1.3145
Pro Ser Thr	0.004368	1.3319
PE(18:4(6Z,9Z,12Z,15Z)/24:1(15Z))	0.000004397	1.1951

a Average of model/average of control.

**Table 3:** The metabolites in untargeted metabolism using LC/MS test under negative detection.

Metabolite	*p*_value	Fold change ^a^
Succinic acid	0.0002778	1.1626
N-Acetyl-L-glutamate 5-semialdehyde	0.00001878	1.1615
Cinncassiol D4 2-glucoside	0.00003857	0.7837
Ganodermic acid Jb	0.000289	0.8018
2-Hydroxyethanesulfonate	0.001307	0.6577
(4-{[2-Methoxy-4-(prop-2-en-1-yl)phenoxy]carbonyl}phenyl)oxidanesulfonic acid	0.001923	0.7603
[3-(6,7-Dihydroxy-4-oxo-4H-chromen-2-yl)phenyl]oxidanesulfonic acid	0.00778	1.4144
Neomacrostemonoside D	0.00641	2.094
Notoginsenoside T1	0.0000725	1.4119
Xanthosine	0.0004708	1.5033
Phosphoserine	0.00001228	1.4318
Caffeic acid	0.0001431	1.3395
6-(Acetyloxy)-3,4,5-trihydroxyoxane-2-carboxylic acid	0.001137	1.2628
{2-Hydroxy-5-[3-(2-hydroxyphenyl)propanoyl]phenyl}oxidanesulfonic acid	0.001133	1.2228
1-(sn-Glycero-3-phospho)-1D-myo-inositol	0.004542	1.3589
2,6-Dihydroxy-4-methoxytoluene	0.0007549	1.4407
4-Methylcatechol	0.0003004	1.2266
6-({8-[(3,3-Dimethyloxiran-2-yl)methyl]-2-oxo-2H-chromen-7-yl}oxy)-3,4,5-trihydroxyoxane-2-carboxylic acid	0.0006586	1.6652
{5-[(E)-2-(3,5-Dihydroxyphenyl)ethenyl]-2-methoxyphenyl}oxidanesulfonic acid	0.0000207	1.4394
Coriandrone D	0.0002286	1.3899
8-Hydroxy-2-methyl-2-(4-methylpent-3-en-1-yl)-2H-chromene-5-carboxylic acid	0.0001316	1.2257
Lipoyllysine	0.00006818	1.2163
L-Prolyl-L-proline	0.00002701	1.6605
Methylmalonic acid	0.0001768	1.1603
Blumealactone C	0.0009278	1.42872
Repaglinide aromatic amine	0.000008321	1.6065
3a,21-Dihydroxy-5b-pregnane-11,20-dione	0.0001108	1.1887
(+/−)11-HDoHE	0.000002086	1.1939
Dihydrogenistein	0.00002944	1.3232
EPIAFZELECHIN (2R,3R)(−)	0.00001128	1.5152
Koenimbine	2.585E-07	1.1947
(S)-2-Azetidinecarboxylic acid	0.00005296	1.3569
CMPF	0.00002644	1.2132
VPGPR enterostatin	0.00001497	0.8936
Crispolide	0.002492	0.8371
Euscaphic acid	0.0004197	0.8706
2-Methoxy-1,4-benzoquinone	0.004925	1.2204
12-Hydroxyicosanoic acid	0.002057	1.1975
Xanthurenic acid	0.0006594	1.2167
Trilobinone	0.0000001749	1.0992
Enterodiol	0.00007841	1.1388
3-Methylorsellini acid	0.0008165	1.178
1-Hydroxy-2-{9-hydroxy-2-oxo-2H,8H,9H-furo[2,3-h]chromen-8-yl}propan-2-yl 3-methylbut-2-enoate	0.009196	1.2205
3,4,5-Trihydroxy-6-(2-hydroxy-1,2-diphenylethoxy)oxane-2-carboxylic acid	0.003854	1.1743
3’-Amino-3′-deoxythimidine glucuronide	0.0007016	1.1665
Portulacaxanthin II	0.003651	1.2026
3,7-Dihydroxyflavone	0.006511	1.1928
LysoPA(8:0/0:0)	0.0001827	1.1618

a Average of model/average of control.

**Figure 4:**
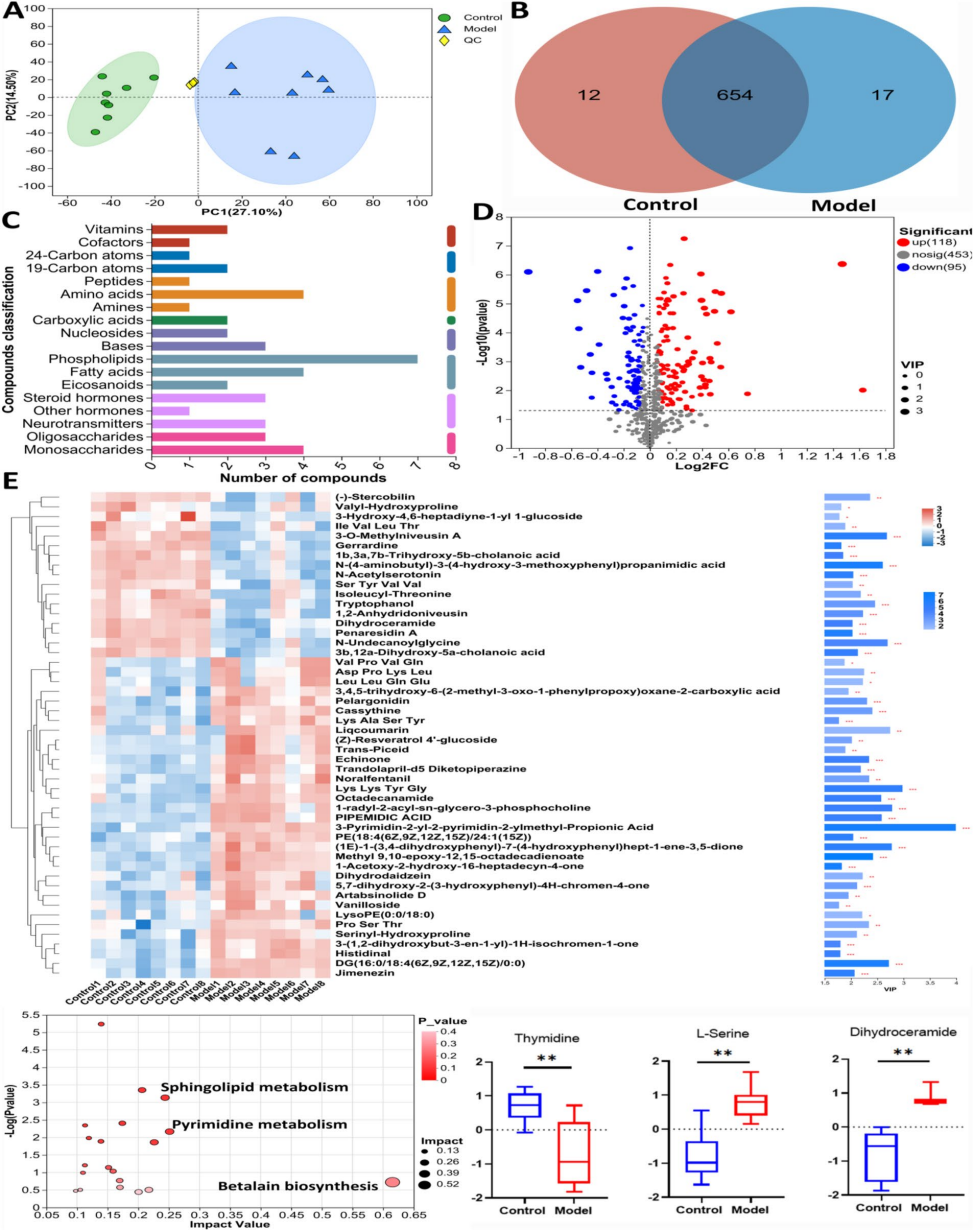
The untargeted metabolism of the feces in mice using LC/MS test under positive detection. **(A)** The PCA plot showed a good separation of the samples in the two groups. **(B)** Venn diagram illustrated the common and differential 683 metabolites between the groups. **(C)** KEGG analysis showed the important pathways involved in the differential metabolites. **(D)** Volcano plot of the 213 differential metabolites between the groups. **(E)** Heatmap diagram showed the change trends of the differential metabolites, and the relative levels of some important metabolites in the critical pathways in Control and Model groups. Data are presented as the mean ± SD (*n* = 8). ^**^p < 0.01, compared with Model group.

Figure 4 showed the results of the positive detection pattern. In Figure 4A, the results of principal component analysis (PCA) showed a significant difference in metabolite levels between Control group and Model group. The results of the Venn diagram (Figure 4B) showed that 654 metabolites were co-existing between Control group and Model group. Figure 4C showed the categorization of the detected metabolites, with phospholipids, amino acid compounds and fatty acid compounds having the highest number of species. The volcano plot (Figure 4D) shows that 118 significantly up-regulated metabolites and 98 significantly down-regulated metabolites were found in Model group compared to Control group (Log_2_FC < 1.5, *p* < 0.05). Figure 4E showed the cluster analysis, statistical analysis and KEGG pathway enrichment analysis of the differential metabolites. L-serine, dihydroceramide levels were significantly increased and thymidine level was significantly decreased in Model mice compared with Control group. The results of pathway enrichment showed that pyrimidine metabolism, sphingolipid metabolism, and betaine biosynthesis were the major metabolic pathways enriched for differential metabolites.

**Figure 5:**
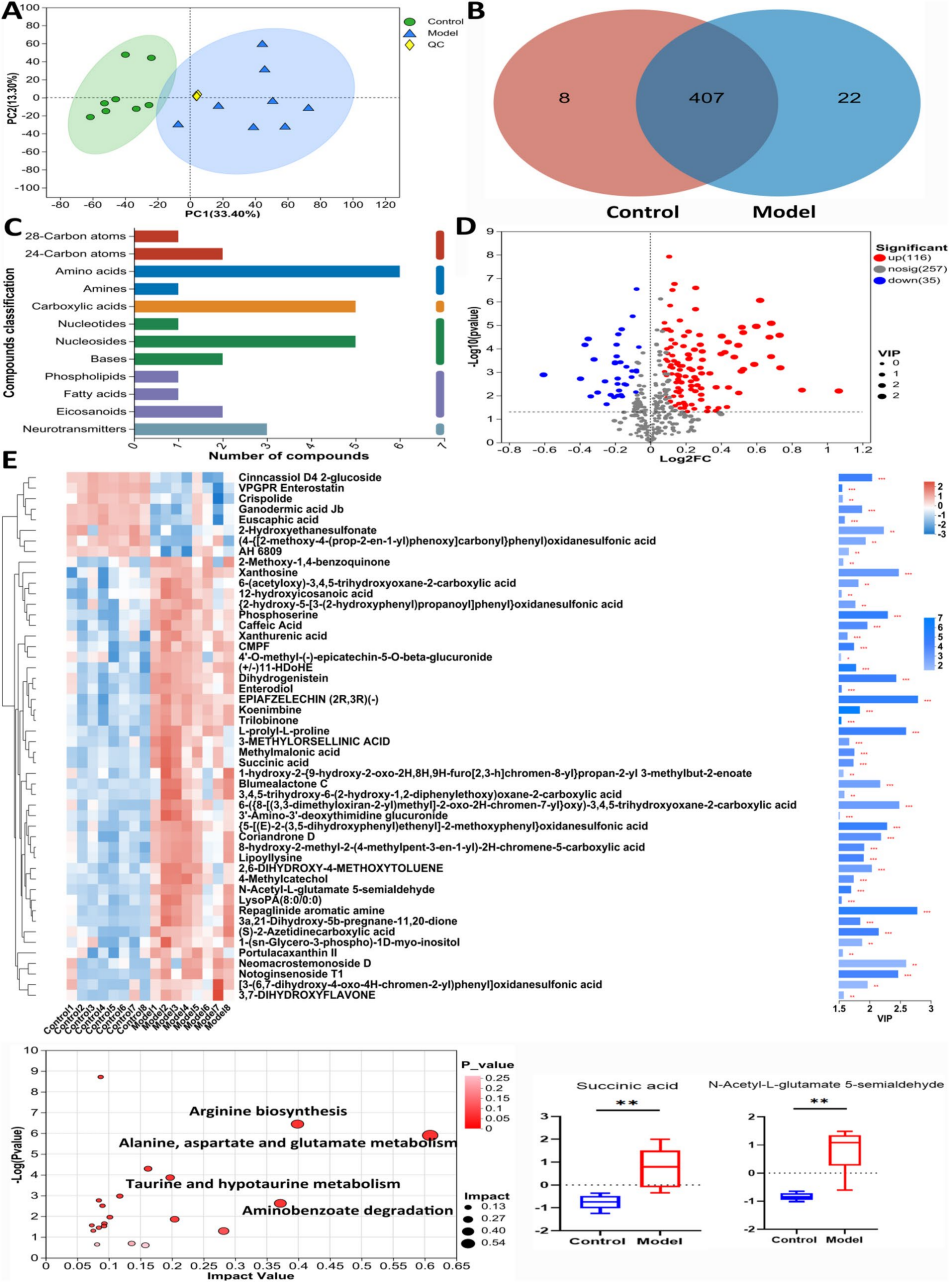
The untargeted metabolism of the feces in mice using LC/MS test under negative detection. **(A)** The PCA plot showed a good separation of the samples in the two groups. **(B)** Venn diagram illustrated the common and differential 437 metabolites between the groups. **(C)** KEGG analysis showed the important pathways involved in the differential metabolites. **(D)** Volcano plot of the 151 differential metabolites between the groups. **(E)** Heatmap diagram showed the change trends of the differential metabolites in Control and Model groups, and the relative levels of some important metabolites in the critical pathways. Data are presented as the mean ± SD (*n* = 8). ^**^p < 0.01 compared with Model group.

Figure 5 showed the results of the negative detection mode. In Figure 5A, the results of principal component analysis (PCA) showed significant differences in metabolite levels between Control and Model groups. The results of the Venn diagram (Figure 5B) showed 407 metabolites co-occurring between Control and Model groups. Figure 5C shows the categorization of the detected metabolites, with amino acids and nucleosides having the highest number of species. The volcano plot (Figure 5D) shows that 116 significantly up-regulated metabolites and 35 significantly down-regulated metabolites were found in Model group compared to Control group (Log_2_FC < 1.5, *p* < 0.05). Figure 5E showed the cluster analysis, statistical analysis and KEGG pathway enrichment analysis of the differential metabolites. Succinate and N-acetylglutamate-5-semialdehyde levels were significantly elevated in Model mice compared with Control. The results of pathway enrichment showed that alanine, aspartate and glutamate metabolism, aminobenzoate degradation, and arginine biosynthesis were the major metabolic pathways enriched for the differential metabolites.

### 3.4 Correlation of bacteria and metabolites

**Figure 6:**
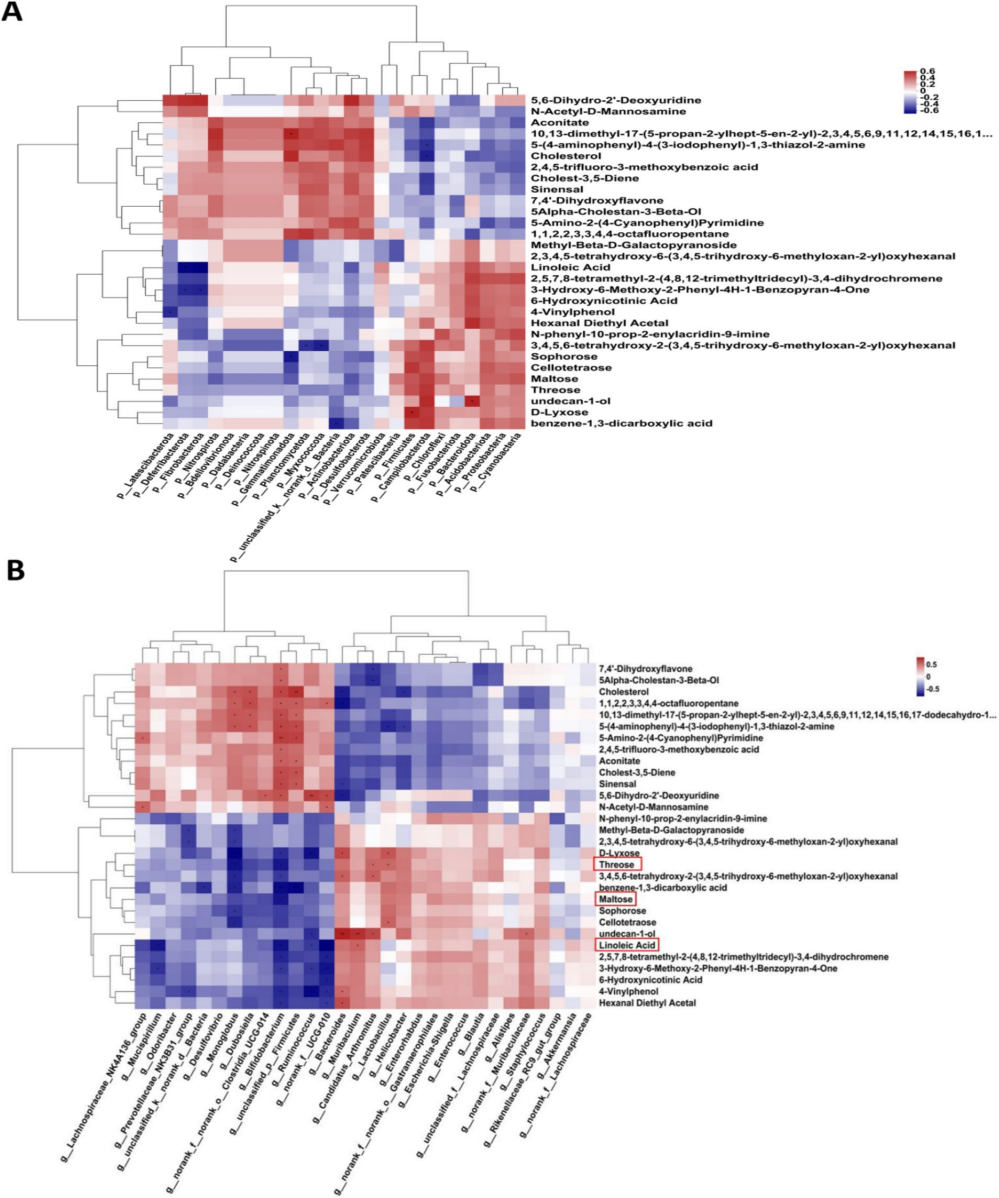
The correlation analysis between the bacteria and the metabolites using GC/MS. **(A)** The correlation analysis on phylum level. **(B)** The correlation analysis on the genus level.

As shown in Figure 6, the results at the genus level revealed a negative correlation between linoleic acid and the abundances of *Bifidobacterium* and *Ruminococcus*. Additionally, *Bifidobacterium* was negatively correlated with maltose and threonine levels. These findings suggest a potential association between linoleic acid, trehalose, and maltose and the occurrence of AD mediated by the abundances of *Bifidobacterium* and *Ruminococcus*.

### 3.5 Relationship between targeted metabolites and bacteria

**Figure 7:**
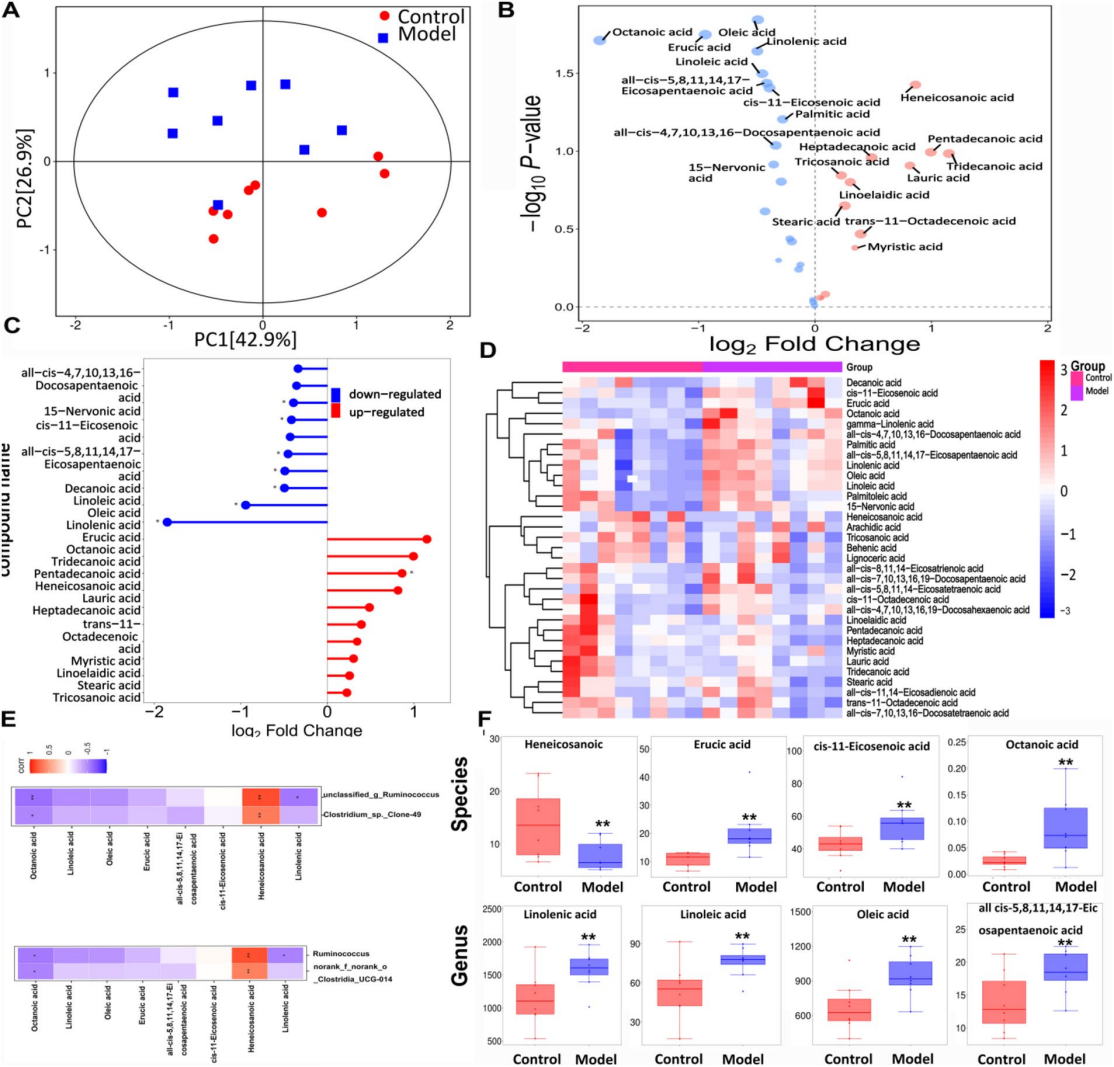
The targeted free fatty acids metabolism of the feces in mice using GC- TOF/MS. **(A)** OPLS-DA score scatter plot showed that all samples were within the 95% confidence interval. **(B)** Volcano plot of 25 free fatty acids metabolites between the groups. **(C)** Matchstick analysis displayed the highly variable differential metabolites between the groups. **(D)** Heatmap diagram showed the change trends of the differential free fatty acids metabolites in Control and Model groups. **(E)** Correlation analysis between the bacteria on genus level and free fatty acids metabolites. **(F)** The levels of free fatty acids metabolites. Data are presented as the mean ± SD (*n* = 8). ^*^p < 0.05, ^**^p < 0.01 compared with Model group.

Figure 7A shows that the samples from the two groups were well separated. The volcano plot depicted in Figure 7B illustrates the trends in the metabolites that exhibited notable differences between the two groups, with red indicating upward trends and blue indicating downward trends. Figure 7C shows the magnitude of the changes in the identified differential metabolites. Figure 7D shows a hierarchical clustering plot of these metabolites. The correlation analysis between the differentially expressed bacteria and metabolites at the genus and species levels is shown in Figure 7E, revealing a negative correlation between *Ruminococcus* and linolenic acid at both genus and species levels. Figure 7F shows a histogram depicting the changes in the abundance of highly variable compounds. Further details regarding other differential metabolites are shown in Table 4.

**Table 4:** The metabolites in targeted metabolism test using GC/MS test.

Compound name	*p*-value	Fold change ^a^
Linoleic acid	0.03182660308	1.369681063
Linolenic acid	0.02282498767	1.410104694
all-cis-4,7,10,13,16-Docosapentaenoic acid	0.09184240582	1.264109293
cis-11-Eicosenoic acid	0.03933066957	0.961050601
all-cis-5,8,11,14,17-Eicosapentaenoic acid	0.03662481898	1.162624122
Oleic acid	0.01434018035	1.405445357
Heneicosanoic acid	0.03748045794	0.549860777
Octanoic acid	0.01947462694	3.603650822
Decanoic acid	0.24348939762	1.346758148
Lauric acid	0.12376195196	0.568101732
Tridecanoic acid	0.103583486	0.450519563
Myristic acid	0.41717174681	0.78772555
Pentadecanoic acid	0.10158119480	0.501779299
Palmitic acid	0.06250595096	1.212784406
Palmitoleic acid	0.57386467588	1.102480472
Heptadecanoic acid	0.10993872844	0.713848405
Stearic acid	0.22391163622	0.83734161
trans-11-Octadecenoic acid	0.34026031391	0.761752297
cis-11-Octadecenoic acid	0.87657299008	0.961050601
Linoelaidic acid	0.15819234966	0.810827421
gamma-Linolenic acid	0.15701154985	1.222941802
Arachidic acid	0.53575929715	1.091090568
all-cis-11,14-Eicosadienoic acid	0.98287496200	1.003657461
all-cis-8,11,14-Eicosatrienoic acid	0.82865489571	0.939981649
all-cis-5,8,11,14-Eicosatetraenoic acid	0.36466758104	1.162624122
Behenic acid	0.89638776236	1.015132239
Tricosanoic acid	0.14303891493	0.855286604
all-cis-7,10,13,16-Docosatetraenoic acid	0.87342263425	0.971947276
Lignoceric acid	0.93058593854	1.009857392
all-cis-7,10,13,16,19-Docosapentaenoic acid	0.50217832471	1.2424553
15-Nervonic acid	0.12188358394	1.279572203
all-cis-4,7,10,13,16,19-Docosahexaenoic acid	0.38076577423	1.264109293

a Average of model/average of control.

### 3.6 Fecal transplantation relieved the symptoms of AD mice

**Figure 8:**
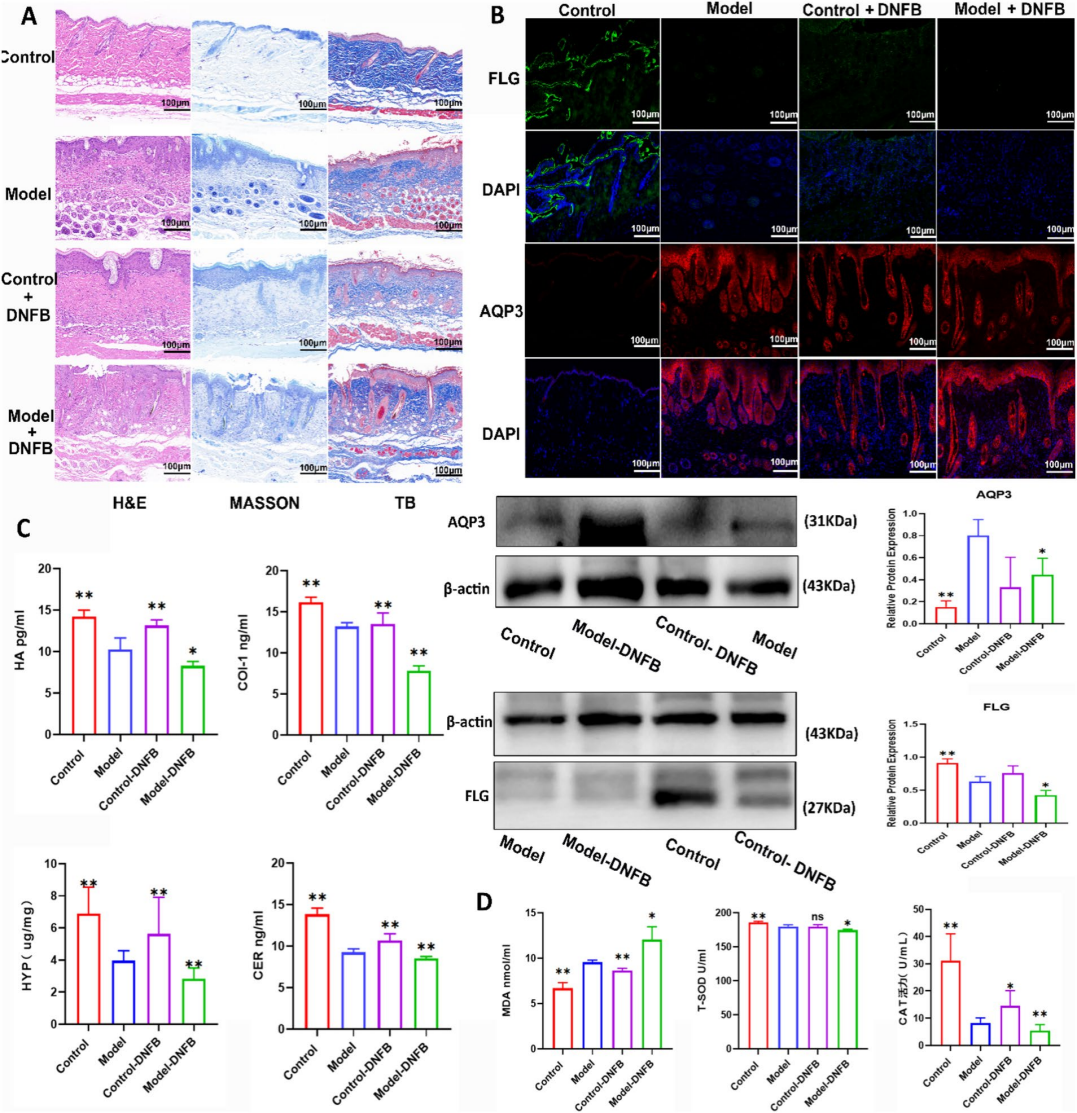
Fecal microbiota transplantation alleviated atopic dermatitis in mice. **(A)** The data of HE, Masson, and toluidine blue staining in mice after FMT on AD. **(B)** FMT changed the expression levels of AQP3 and FLG in AD mice by western blot and immunofluorescence tests. Data are presented as the mean ± SD (*n* = 3). **(C)** FMT reversed the levels of COL I, HA, CER and HYP in AD mice. Data are presented as the mean ± SD (*n* = 5). **(D)** FMT reversed the levels of T-SOD, MDA and CAT in mice AD. Data are presented as the mean ± SD (*n* = 5). ^*^p < 0.05, ^**^p < 0.01 compared with Model group.

In Figure 8A, based on hematoxylin and eosin, Masson trichrome and toluidine blue assays, mice in Model + DNFB group had the most severe skin damage. As shown in Figure 8B, the results of immunofluorescence and Western blotting tests showed that FLG levels were decreased and AQP3 levels were increased in Model + DNFB group compared with Model group. Figure 8C showed that the levels of HA, COI-1, HYP and CER were decreased in Model + DNFB group compared with Model group. In Figure 8D, the levels of MDA were obviously elevated and the levels of T-SOD and CAT were significantly decreased in Model + DNFB group compared with Model group. However, all of these indicators were significantly improved in the Control + DNFB group, confirming that AD symptoms were improved by transplanting colonies from healthy mice.

## 4 Discussion

Atopic Dermatitis (AD) is a common chronic, recurrent, inflammatory skin disease. Its symptoms are mainly inflammation and flaking of the skin, erythema, accompanied by an intense itching sensation. The human gut microbiota is a complex and interconnected system that plays a crucial role in human health. The gut microbiota is closely associated with a wide range of chronic diseases and poses a significant risk to human health (Chen et al., 2021). Factors such as the body’s immune response, environmental influences and genetic predisposition combine to determine the structure and composition of the gut microbiota. Therefore, the maintenance of intestinal homeostasis is closely related to the normal functioning of body systems and microbiota (Kataoka, 2016). Microbial metabolites are important mediators of interactions between the microbiota and host cells (Petrosino et al., 2009), highlighting the importance of regulating gut flora and microbiota metabolism in the prevention and treatment of diseases such as AD. With intensive research, many studies have revealed the correlation between gut microbiota and AD. The “gut-skin” axis has been proposed and recognized as a new target for the prevention and treatment of AD (Fang et al., 2021).

In this study, we chose C57BL/6J mice for the experiments, which have similar symptoms to humans. AD mouse models have erythema, itching, and dryness similar to those of humans, which makes them the ideal models for studying AD (Nagata et al., 2021). In animal experiments, 0.5% DNFB was used for model establishment. The results of H&E staining showed that the skin of mice in Model group showed thickening and disorganization of collagen fiber bundles, suggesting that the skin barrier was severely damaged. AQP3 (aquaporin 3) and FLG (filamentous polyglutamic protein) are two important proteins in skin health, which play key roles in skin barrier function and moisturization. In AD patients, AQP3 expression level is significantly increased. This may be due to the decreased epidermal water content in patients with chronic eczema, and the epidermal stratum corneum cells compensatory express large amounts of AQP3 to facilitate the penetration of more water and glycerol from the body into the epidermal stratum corneum (Lu et al., 2023). In contrast, FLG expression is reduced in skin lesions in AD patients (Guttman-Yassky et al., 2019). In our work, the expression levels of these two proteins in the skin were examined by immunofluorescence and Western tests. FLG levels were significantly decreased and AQP3 levels were significantly increased in the skin tissues of Model mice compared to Control group. AD also triggers inflammation and oxidative stress (Huang et al., 2022; Bertino et al., 2020). We examined three common oxidative stress indicators (T-SOD, MDA, CAT) and three inflammatory factors (IL-1β, IL-6, TNF-*α*). The results showed that the levels of T-SOD and CAT were significantly decreased and the levels of MDA, IL-1β, IL-6, and TNF-α were significantly increased in Model mice compared with Control group. Hydroxyproline, hyaluronic acid, collagen I, and ceramides are several substances that have important roles in reducing the pathogenesis of AD. The experimental results showed that all four substances were significantly decreased in the skin tissues of mice in Model group compared to Control group. In conclusion, all the data indicate that the mouse modeling for sequencing was very successful.

16S rRNA gene sequencing method can provide valuable information for understanding bacterial composition (Johnson et al., 2019). In the present study, we observed significant changes in the composition of microorganisms associated with AD. In particular, *Bifidobacterium* and *Ruminococcus*. The results of this study showed that the abundances of *Bifidobacterium* and *Ruminococcus* were significantly decreased in Model mice compared to Control group. *Bifidobacterium* has been widely used in food and health care as the recognized probiotic. Fang et al. have found that *Bifidobacterium longum* can mediate tryptophan metabolism to ameliorate atopic dermatitis through the gut-skin axis (Fang et al., 2022). The exact mechanism regarding the association of *Ruminococcus* with the pathogenesis of atopic dermatitis has not been accurately verified. Mari Sasaki et al. have found that the abundance of *Ruminococcus bromii* was correlated with fecal butyrate levels and atopic dermatitis in infants (Sasaki et al., 2022). Jae-Rin Ahn et al. have also found in animal experiments that *Ruminococcus* improves atopic dermatitis by enhancing the Treg cells and metabolites of BALB/c mice (Ahn et al., 2022). In conclusion, the abundance of *Bifidobacteria* and *Ruminococcus* were significantly reduced after the onset of AD, and restoring their levels should improve the symptoms of AD.

When the flora is altered, the level of metabolism in the organism is also altered. Metabolomics provides a powerful tool for identifying biomarkers and gaining insight into disease mechanisms, thus facilitating the development of precision medicine approaches (Cui et al., 2018). Our study identified different metabolites using GC-TOF/MS and UPLC-MS/MS analyses, revealing their association with specific intestinal bacteria at different levels of classification. The experimental results showed that the metabolites such as linoleic acid, maltose, threonine thymine, L-serine, dihydroceramide, succinate and N-acetylglutamate-5-semialdehyde were significantly elevated in the model group as compared to the control group. Topical application of linoleic acid usually provides relief from atopic dermatitis. However, linoleic acid metabolism levels were significantly elevated *in vivo* in this study. We were intrigued by this phenomenon, and thus we further performed a targeted metabolomics assay, i.e., absolute quantification of the class of free fatty acids in mouse feces. The results were consistent with the untargeted results in that linoleic acid levels were significantly elevated in the model mice. We hypothesized that this may be some kind of *in vivo* protective mechanism to promote linoleic acid metabolism, but this speculation remains to be verified. To verify the potential link between the genera *Ruminococcus* and *Bifidobacterium* and linoleic acid, we performed genus-metabolite correlation analyses. The results of the joint assay showed that linoleic acid was significantly negatively correlated with *Ruminococcus* and *Bifidobacterium*. That was, the metabolic level of linoleic acid showed the increased tendency when the abundances of *Ruminococcus* and *Bifidobacterium* decreased, suggesting that *Ruminococcus* and *Bifidobacterium* may be involved in linoleic acid metabolism.

Fecal microbiota transplantation experiments demonstrated that mice receiving fecal transplants from Control + DNFB group exhibited reduced AD symptoms compared with those receiving transplants from Model + DNFB group. These improvements were accompanied by reductions in skin barrier damage and AQP3 levels, as well as elevations in FLG levels, underscoring the potential therapeutic benefits of modulating the gut microbiota composition (Smits et al., 2013).

This study discovered a number of bacteria and metabolites with significant changes caused by AD. In particular, the differential genera screened, *Ruminococcus* and *Bifidobacterium*, should be possible association with linoleic acid metabolism *in vivo* for the first time. These data provided experimental basis for in-depth research on the pathogenesis of AD and feasible prevention and treatment methods. Identifying specific metabolites and their association with microbial taxa can provide valuable insights into disease mechanisms and potential therapeutic targets. These findings should pave the way for future research to elucidate the underlying mechanisms of AD and develop targeted interventions to improve patient outcomes. The limitation of this study was that the functions of bacteria and metabolites with significant changes on regulating AD were not studied in depth, and in addition, the role and application of the association of bacteria and metabolites were not investigated, and the possible application of the bacteria and metabolites on AD were also not tested, which will be our next research focus. In our future plans, *Ruminococcus* and *Bifidobacterium* will be colonized in germ-free mice to confirm which supplementation is more effective in improving AD. We will also quantify the changes in linoleic acid content in the feces of different groups of mice to verify the relationship between bacteria and metabolites *in vivo.* At the same time, the bacteria will be co-cultured with linoleic acid to validate *in vitro*. Finally, the specific target of action will be screened by transcriptomics, and the mechanism of action of the bacteria will be completely elaborated.

## Data Availability

The original contributions presented in the study are included in the article/Supplementary material, further inquiries can be directed to the corresponding author.

## Footnotes

^1^ https://cloud.majorbio.com

^2^ http://www.hmdb.ca/

^3^ https://metlin.scripps.edu/
