# Supplementary material for: The changes of intestinal flora and metabolites in atopic dermatitis mice
Source: Front Microbiol. 2024 Dec 16;15:1462491. doi: 10.3389/fmicb.2024.1462491 (PMC11683101; doi:10.3389/fmicb.2024.1462491)
Supplement: Supplementary file 1 [file Table_1.docx]

**Supplemental Table S1** The metabolites in untargeted metabolism by GC/MS assay

| Metabolite | P-Value | Fold change ^a^ |
| --- | --- | --- |
| Hexanal diethyl acetal | 0.0001558 | 1.1405 |
| Maltose | 0.001671 | 1.1037 |
| Threose | 0.00131 | 1.074 |
| Linoleic acid | 0.001043 | 1.0499 |
| (4-Methoxyphenoxy)-dimethyl-pentadecoxysilane | 0.00006887 | 1.1573 |
| Aconitate | 0.000001284 | 0.8901 |
| 4-Vinylphenol | 0.00004539 | 1.0906 |
| Maltotriitol | 0.001549 | 1.1485 |
| Cellotetraose | 0.002681 | 1.1256 |
| D-(+)-Trehalose | 0.04235 | 1.1637 |
| Sophorose | 0.004513 | 1.1069 |
| 1,3-Diphenyl-9H-indeno[2,1-c]pyridine | 0.007016 | 1.1283 |
| 2,4,5-Trifluoro-3-methoxybenzoic acid | 0.00000937 | 0.894 |
| 1,1,2,2,3,3,4,4-Octafluoropentane | 0.00000268 | 0.9417 |
| 5,6-Dihydro-2'-deoxyuridine | 0.01658 | 0.884 |
| N-Acetyl-D-mannosamine | 0.009544 | 0.9292 |
| (2,5-dimethylphenoxy)-dimethyl-tetradecoxysilane | 0.003356 | 0.9266 |
| Undecan-1-ol | 0.005061 | 1.1524 |
| 2,3,4,5-Tetrahydroxy-6-(3,4,5-trihydroxy-6-methyloxan-2-yl)oxyhexanal | 0.02848 | 1.0966 |
| Methyl-beta-D-galactopyranoside | 0.02845 | 1.0964 |
| 2,5,7,8-tetramethyl-2-(4,8,12-trimethyltridecyl)-3,4-dihydrochromene | 0.0001583 | 1.0679 |
| 6-Hydroxynicotinic Acid | 0.0006661 | 1.0659 |
| 3-Hydroxy-6-methoxy-2-phenyl-4H-1-benzopyran-4-one | 0.001494 | 1.0568 |
| Benzene-1,3-dicarboxylic acid | 0.005963 | 1.0641 |
| D-Lyxose | 0.0005913 | 1.05 |
| Diethyl(dihexoxy)silane | 0.008925 | 1.1247 |
| Melezitose | 0.005494 | 1.1071 |
| 3,4,5,6-Tetrahydroxy-2-(3,4,5-trihydroxy-6-methyloxan-2-yl)oxyhexanal | 0.003902 | 1.0906 |
| 5-Sulfosalicylate | 0.01058 | 1.2336 |
| N-phenyl-10-prop-2-enylacridin-  9-Imine | 0.0105 | 1.1319 |
| 9-Undecylanthracene | 0.0006455 | 0.8196 |
| 1,4-Dideoxy-1,4-imino-d-arabinitol | 0.001379 | 0.9423 |
| Sinensal | 0.0000289 | 0.9376 |
| Cholest-3,5-diene | 0.0000585 | 0.945 |
| Dodecoxy-(4-methoxyphenoxy)-dimethylsilane | 0.0000105 | 0.9225 |
| hexadecanoic acid | 0.0003861 | 0.8392 |
| (5S,8R,9S,10S,13R,14S,17R)-10,13-dimethyl-17-[(2R)-6-methylheptan-2-yl]-2-phenylsulfanyl-1,2,4,5,6,7,8,9,11,12,14,15,16,17-tetradecahydrocyclopenta[a]phenanthren-3-one | 0.00000931 | 0.8306 |
| 5-(4-Aminophenyl)-4-(3-iodophenyl)-1,3-thiazol-2-amine | 0.0000127 | 0.9392 |
| 5,7-Dimethylpyrimido(1,6-a)indole | 0.000000801 | 0.7309 |
| 10,13-Dimethyl-17-(6-methylhept-5-en-2-yl)-2,3,4,7,8,9,11,12,14,15,16,17-dodecahydro-1H-cyclopenta[a]phenanthren-3-ol | 0.0006366 | 0.7883 |
| 5Alpha-cholestan-3-beta-ol | 0.0009802 | 0.7542 |
| 7,4'-Dihydroxyflavone | 0.001275 | 0.7765 |
| 5-Amino-2-(4-cyanophenyl)pyrimidine | 0.000094 | 0.9307 |
| 10,13-Dimethyl-17-(5-propan-2-ylhept-5-en-2-yl)-2,3,4,5,6,9,11,12,14,15,16,17-dodecahydro-1H-cyclopenta[a]phenanthren-3-ol | 0.0003687 | 0.9537 |
| 1-(2-Bromoethylsulfonyl)butane | 0.01468 | 0.8691 |
| 1-(Carboxyamino)cyclopentane-1-carboxylic acid | 0.001489 | 0.94 |
| Methyl linolenate | 0.0149 | 0.8713 |
| 3,5-Bis(3-methoxyphenyl)-2,3-dihydroinden-1-one | 0.01299 | 0.8446 |
| Oleyl amide | 0.0001217 | 0.6712 |
| Cholesterol | 0.0000246 | 0.9662 |

a Average of Model/Average of Control

Supplemental Table S2 The differential metabolites in untargeted metabolism by LC/MS test under positive detection

| Metabolite | P_value | Fold change ^a^ |
| --- | --- | --- |
| Dihydroceramide | 0.0000313 | 0.8671 |
| Lys Lys Tyr Gly | 0.00000768 | 1.3147 |
| Thymidine | 0.002491 | 1.1461 |
| L-Serine | 0.000104 | 0.879 |
| N-Undecanoylglycine | 0.0000736 | 0.6866 |
| (-)-Stercobilin | 0.002687 | 0.7945 |
| 3-Pyrimidin-2-yl-2-pyrimidin-2-ylmethyl-  Propionic acid | 0.000000426 | 2.7699 |
| 1-Radyl-2-acyl-sn-glycphocholinero-3-phose | 0.0000192 | 1.5367 |
| 3b,12a-Dihydroxy-5a-cholanoic acid | 0.0000122 | 0.8729 |
| (Z)-Resveratrol 4'-glucoside | 0.001392 | 1.2159 |
| 1-Acetoxy-2-hydroxy-16-heptadecyn-4-one | 0.0000004585 | 1.1141 |
| 3-(1,2-Dihydroxybut-3-en-1-yl)-1H-isochromen-1-one | 0.0000701 | 1.1302 |
| 5,7-Dihydroxy-2-(3-hydroxyphenyl)-4H-chromen-4-one | 0.000773 | 1.2531 |
| Wogonin | 0.009406 | 1.1466 |
| 1b,3a,7b-Trihydroxy-5b-cholanoic acid | 0.0000331 | 0.8957 |
| Methyl 9,10-epoxy-12,15-octadecadienoate | 0.0000000564 | 1.1993 |
| Noralfentanil | 0.001128 | 1.3219 |
| Alanyl-glutamic acid | 0.007024 | 1.1662 |
| Asparaginyl-glutamic acid | 0.001311 | 1.1603 |
| Norepinephrine (noradrenaline) | 0.000007838 | 1.1055 |
| 5-Hydroxymethyluracil | 0.001585 | 1.1863 |
| 10-Hydroxymyristic acid methyl ester | 0.001353 | 0.8927 |
| Asp Pro Lys Leu | 0.008003 | 1.3047 |
| N-Acetyl-7-O-acetylneuraminic acid | 0.005302 | 1.1881 |
| Tryptophanol | 0.0005714 | 0.7295 |
| Trans-Piceid | 0.002168 | 1.1946 |
| Pelargonidin | 0.0004757 | 1.265 |
| Octadecanamide | 0.0000143 | 1.3349 |
| 9,10,13-TriHOME | 0.0000215 | 1.1151 |
| Ile Val Leu Thr | 0.007643 | 0.817 |
| Penaresidin A | 0.000001347 | 0.8842 |
| DG(16:0/18:4(6Z,9Z,12Z,15Z)/0:0) | 0.0000009399 | 1.3105 |
| Jimenezin | 0.000005596 | 1.1335 |
| 3-beta-Hydroxy-4-beta-methyl-5-alpha-cholest-7-ene-4-alpha-carboxylate | 0.000488 | 0.894 |
| 3-Buten-2-one 1-(2,3,6-trimethyl phenyl) | 0.00007061 | 0.8862 |
| N-Acetyltyramine | 0.0006793 | 0.8898 |
| LysoPC(22:6(4Z,7Z,10Z,13Z,16Z,19Z)) | 0.005064 | 0.8883 |
| 5,8-Epoxy-5,8-dihydro-3-hydroxy-8'-apo-b,y-carotenal | 0.002153 | 1.1332 |
| 3-O-Methylniveusin A | 0.000003551 | 0.7155 |
| (1E)-1-(3,4-dihydroxyphenyl)-7-(4-hydroxyphenyl)hept-1-ene-3,5-dione | 0.0000044 | 1.4604 |
| Dihydrodaidzein | 0.006332 | 1.3421 |
| Trandolapril-d5 diketopiperazine | 0.0001495 | 1.2226 |
| Liqcoumarin | 0.009832 | 3.0888 |
| Isoleucyl-Threonine | 0.002471 | 0.736 |
| Cassythine | 0.0007759 | 1.3748 |
| Lys Ala Ser Tyr | 0.0003774 | 1.1577 |
| Vanilloside | 0.001919 | 1.1992 |
| 3,4,5-Trihydroxy-6-(2-methyl-3-oxo-1-phenylpropoxy)oxane-2-carboxylic acid | 0.007912 | 1.3424 |
| N-Acetylserotonin | 0.000005031 | 0.8258 |
| Tetradecanedioic acid | 0.003833 | 0.8508 |
| N-(4-aminobutyl)-3-(4-hydroxy-3-methoxyphenyl)propanimidic acid | 0.0000007698 | 0.7586 |
| Gerrardine | 0.000002907 | 0.8736 |
| Ser Tyr Val Val | 0.004217 | 0.7984 |
| 1,2-Anhydridoniveusin | 0.0002604 | 0.7643 |
| Artabsinolide D | 0.001243 | 1.2295 |
| Hypoglycin B | 0.003568 | 1.1492 |
| PhistidinalIpemidic acid | 0.00001845 | 1.4087 |
| Histidinal | 0.00002159 | 1.1312 |
| Serinyl-Hydroxyproline | 0.003301 | 1.3145 |
| Pro Ser Thr | 0.004368 | 1.3319 |
| PE(18:4(6Z,9Z,12Z,15Z)/24:1(15Z)) | 0.000004397 | 1.1951 |

a Average of Model/Average of Control

**Supplemental Table S3** The metabolites in untargeted metabolism by LC/MS test under negative detection

| Metabolite | | P_value | Fold change ^a^ | |
| --- | --- | --- | --- | --- |
| Succinic acid | 0.0002778 | | | 1.1626 |
| N-Acetyl-L-glutamate 5-semialdehyde | 0.00001878 | | | 1.1615 |
| Cinncassiol D4 2-glucoside | 0.00003857 | | | 0.7837 |
| Ganodermic acid Jb | 0.000289 | | | 0.8018 |
| 2-Hydroxyethanesulfonate | 0.001307 | | | 0.6577 |
| (4-{[2-Methoxy-4-(prop-2-en-1-yl)phenoxy]carbonyl}phenyl)oxidanesulfonic acid | 0.001923 | | | 0.7603 |
| [3-(6,7-Dihydroxy-4-oxo-4H-chromen-2-yl)phenyl]oxidanesulfonic acid | 0.00778 | | | 1.4144 |
| Neomacrostemonoside D | 0.00641 | | | 2.094 |
| Notoginsenoside T1 | 0.0000725 | | | 1.4119 |
| Xanthosine | 0.0004708 | | | 1.5033 |
| Phosphoserine | 0.00001228 | | | 1.4318 |
| Caffeic acid | 0.0001431 | | | 1.3395 |
| 6-(Acetyloxy)-3,4,5-trihydroxyoxane-2-carboxylic acid | 0.001137 | | | 1.2628 |
| {2-Hydroxy-5-[3-(2-hydroxyphenyl)propanoyl]phenyl}oxidanesulfonic acid | 0.001133 | | | 1.2228 |
| 1-(sn-Glycero-3-phospho)-1D-myo-inositol | 0.004542 | | | 1.3589 |
| 2,6-Dihydroxy-4-methoxytoluene | 0.0007549 | | | 1.4407 |
| 4-Methylcatechol | 0.0003004 | | | 1.2266 |
| 6-({8-[(3,3-Dimethyloxiran-2-yl)methyl]-2-oxo-2H-chromen-7-yl}oxy)-3,4,5-trihydroxyoxane-2-carboxylic acid | 0.0006586 | | | 1.6652 |
| {5-[(E)-2-(3,5-Dihydroxyphenyl)ethenyl]-2-methoxyphenyl}oxidanesulfonic acid | 0.0000207 | | | 1.4394 |
| Coriandrone D | 0.0002286 | | | 1.3899 |
| 8-Hydroxy-2-methyl-2-(4-methylpent-3-en-1-yl)-2H-chromene-5-carboxylic acid | 0.0001316 | | | 1.2257 |
| Lipoyllysine | 0.00006818 | | | 1.2163 |
| L-Prolyl-L-proline | 0.00002701 | | | 1.6605 |
| Methylmalonic acid | 0.0001768 | | | 1.1603 |
| Blumealactone C | 0.0009278 | | | 1.42872 |
| Repaglinide aromatic amine | 0.000008321 | | | 1.6065 |
| 3a,21-Dihydroxy-5b-pregnane-11,20-dione | 0.0001108 | | | 1.1887 |
| (+/-)11-HDoHE | 0.000002086 | | | 1.1939 |
| Dihydrogenistein | 0.00002944 | | | 1.3232 |
| EPIAFZELECHIN (2R,3R)(-) | 0.00001128 | | | 1.5152 |
| Koenimbine | 2.585E-07 | | | 1.1947 |
| (S)-2-Azetidinecarboxylic acid | 0.00005296 | | | 1.3569 |
| CMPF | 0.00002644 | | | 1.2132 |
| VPGPR enterostatin | 0.00001497 | | | 0.8936 |
| Crispolide | 0.002492 | | | 0.8371 |
| Euscaphic acid | 0.0004197 | | | 0.8706 |
| 2-Methoxy-1,4-benzoquinone | 0.004925 | | | 1.2204 |
| 12-Hydroxyicosanoic acid | 0.002057 | | | 1.1975 |
| Xanthurenic acid | 0.0006594 | | | 1.2167 |
| Trilobinone | 0.0000001749 | | | 1.0992 |
| Enterodiol | 0.00007841 | | | 1.1388 |
| 3-Methylorsellini acid | 0.0008165 | | | 1.178 |
| 1-Hydroxy-2-{9-hydroxy-2-oxo-2H,8H,9H-furo[2,3-h]chromen-8-yl}propan-2-yl 3-methylbut-2-enoate | 0.009196 | | | 1.2205 |
| 3,4,5-Trihydroxy-6-(2-hydroxy-1,2-diphenylethoxy)oxane-2-carboxylic acid | 0.003854 | | | 1.1743 |
| 3'-Amino-3'-deoxythimidine glucuronide | 0.0007016 | | | 1.1665 |
| Portulacaxanthin II | 0.003651 | | | 1.2026 |
| 3,7-Dihydroxyflavone | 0.006511 | | | 1.1928 |
| LysoPA(8:0/0:0) | 0.0001827 | | | 1.1618 |

a Average of Model/Average of Control

**Supplemental Table S4** The metabolites in targeted metabolism test by GC/MS test

| Compound name | Fold change ^a^ | P-value |
| --- | --- | --- |
| Linoleic acid | 1.369681063 | 0.03182660308 |
| Linolenic acid | 1.410104694 | 0.02282498767 |
| all-cis-4,7,10,13,16-Docosapentaenoic acid | 1.264109293 | 0.09184240582 |
| cis-11-Eicosenoic acid | 0.961050601 | 0.03933066957 |
| all-cis-5,8,11,14,17-Eicosapentaenoic acid | 1.162624122 | 0.03662481898 |
| Oleic acid | 1.405445357 | 0.01434018035 |
| Heneicosanoic acid | 0.549860777 | 0.03748045794 |
| Octanoic acid | 3.603650822 | 0.01947462694 |
| Decanoic acid | 1.346758148 | 0.24348939762 |
| Lauric acid | 0.568101732 | 0.12376195196 |
| Tridecanoic acid | 0.450519563 | 0.103583486 |
| Myristic acid | 0.78772555 | 0.41717174681 |
| Pentadecanoic acid | 0.501779299 | 0.10158119480 |
| Palmitic acid | 1.212784406 | 0.06250595096 |
| Palmitoleic acid | 1.102480472 | 0.57386467588 |
| Heptadecanoic acid | 0.713848405 | 0.10993872844 |
| Stearic acid | 0.83734161 | 0.22391163622 |
| trans-11-Octadecenoic acid | 0.761752297 | 0.34026031391 |
| cis-11-Octadecenoic acid | 0.961050601 | 0.87657299008 |
| Linoelaidic acid | 0.810827421 | 0.15819234966 |
| gamma-Linolenic acid | 1.222941802 | 0.15701154985 |
| Arachidic acid | 1.091090568 | 0.53575929715 |
| all-cis-11,14-Eicosadienoic acid | 1.003657461 | 0.98287496200 |
| all-cis-8,11,14-Eicosatrienoic acid | 0.939981649 | 0.82865489571 |
| all-cis-5,8,11,14-Eicosatetraenoic acid | 1.162624122 | 0.36466758104 |
| Behenic acid | 1.015132239 | 0.89638776236 |
| Tricosanoic acid | 0.855286604 | 0.14303891493 |
| all-cis-7,10,13,16-Docosatetraenoic acid | 0.971947276 | 0.87342263425 |
| Lignoceric acid | 1.009857392 | 0.93058593854 |
| all-cis-7,10,13,16,19-Docosapentaenoic acid | 1.2424553 | 0.50217832471 |
| 15-Nervonic acid | 1.279572203 | 0.12188358394 |
| all-cis-4,7,10,13,16,19-Docosahexaenoic acid | 1.264109293 | 0.38076577423 |

a Average of Model/Average of Control

**Supplemental Table S5** Primer sequences of RT-PCR assay

| Gene | Reverse primer (5′-3 ′) | Forward primer (5′-3′) |
| --- | --- | --- |
| GAPDH | GGAAGGCCATGCCAGTGA | GCCACCCAGAAGACTGTGGAT |
| IL-6 | CAGATTGTTTTCTGCAAGTGCAT | ACCACTCCCAACAGACCTGTCT |
| TNF-α | TGGGCTCATACCAGGGTTTG | ACAAGGCTGCCCCGACTAC |


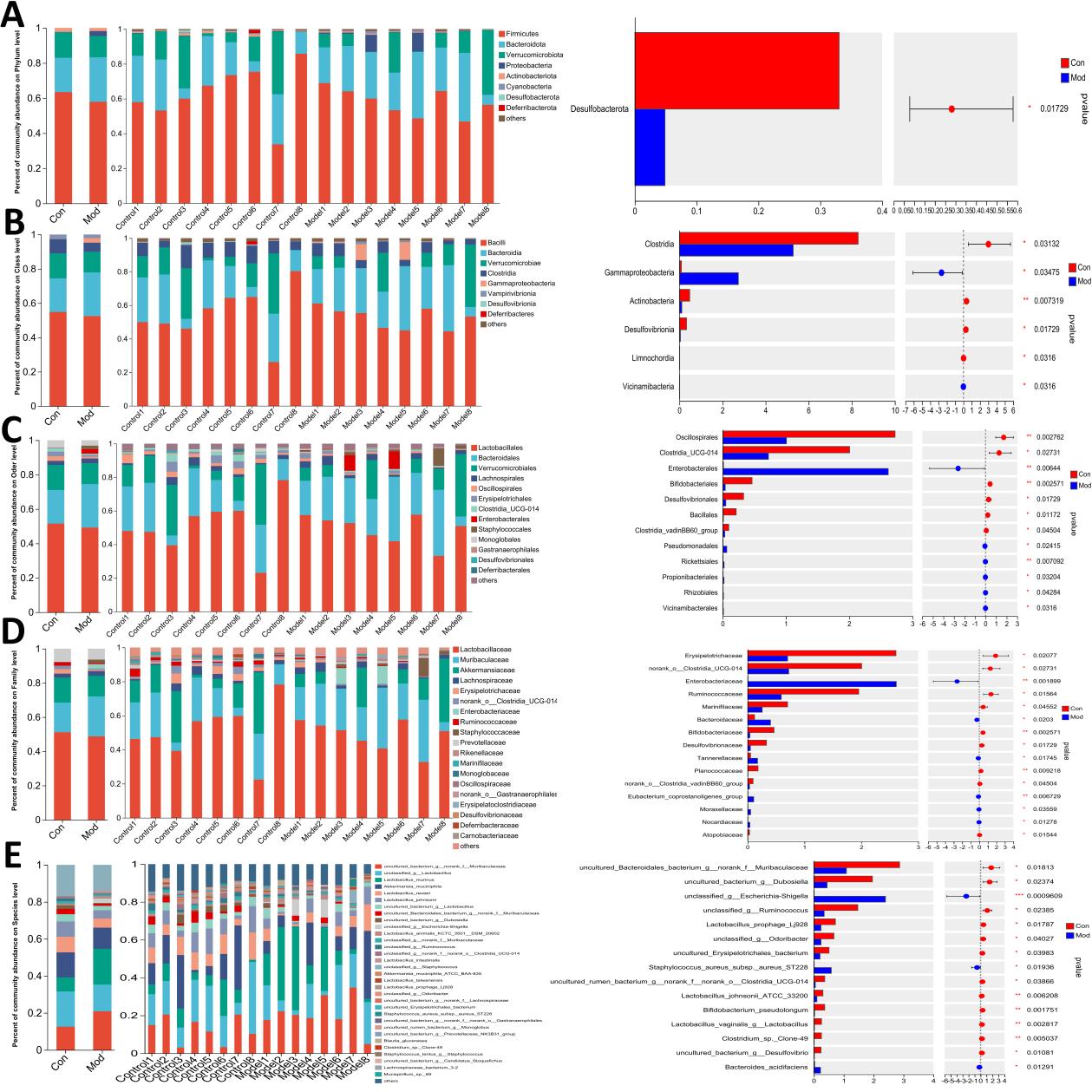


**Supplemental Figure S1.** The histogram of enteric bacteria species differences and analysis plot of species differences between the groups. (A) Phylum level; (B) Class level; (C) Order level; (D) Family level; (E) Species level.


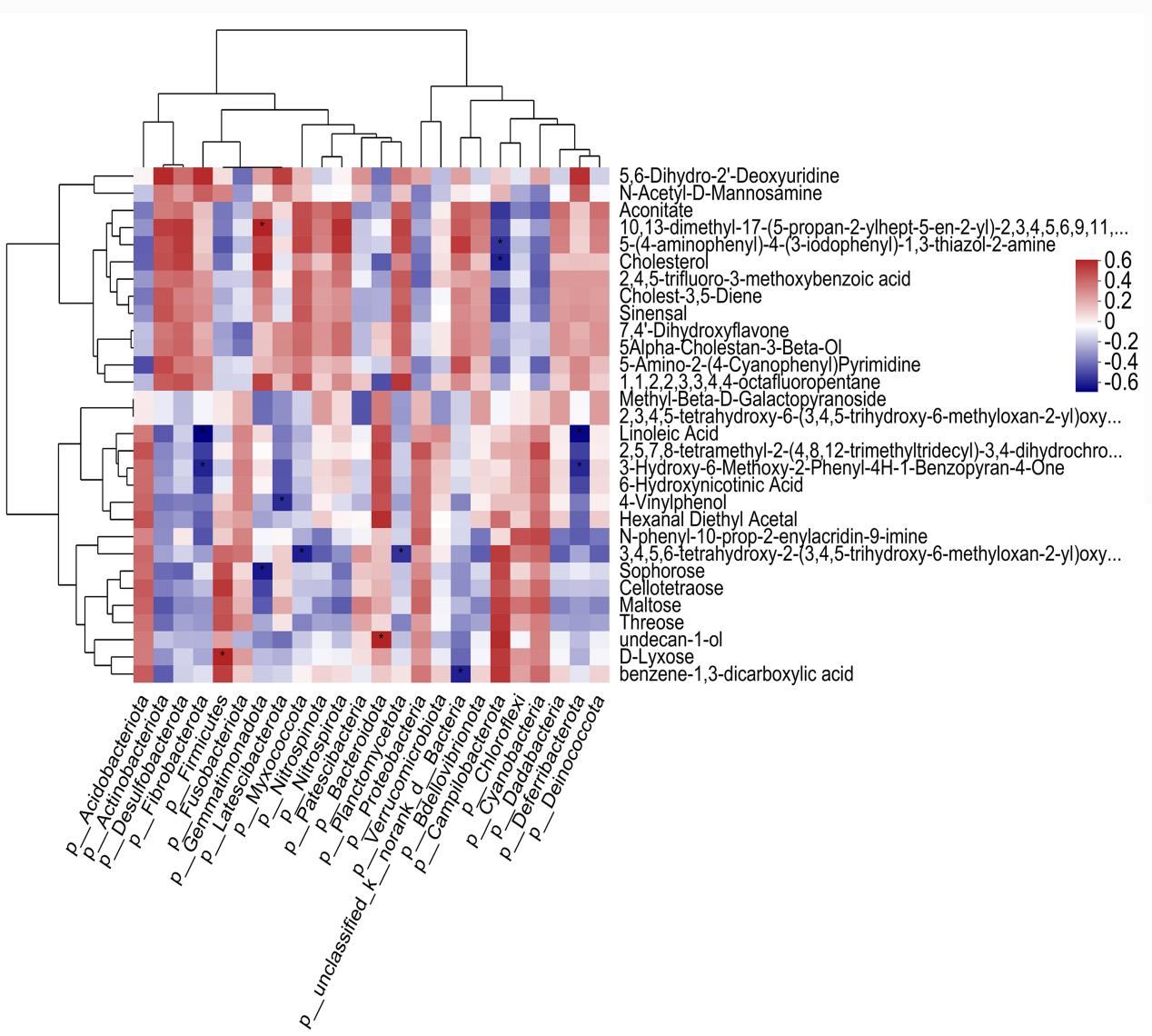


**Supplemental Figure S2.** The correlation analysis of the metabolites under GC-TOF/MS detection with bacteria at plylum level.


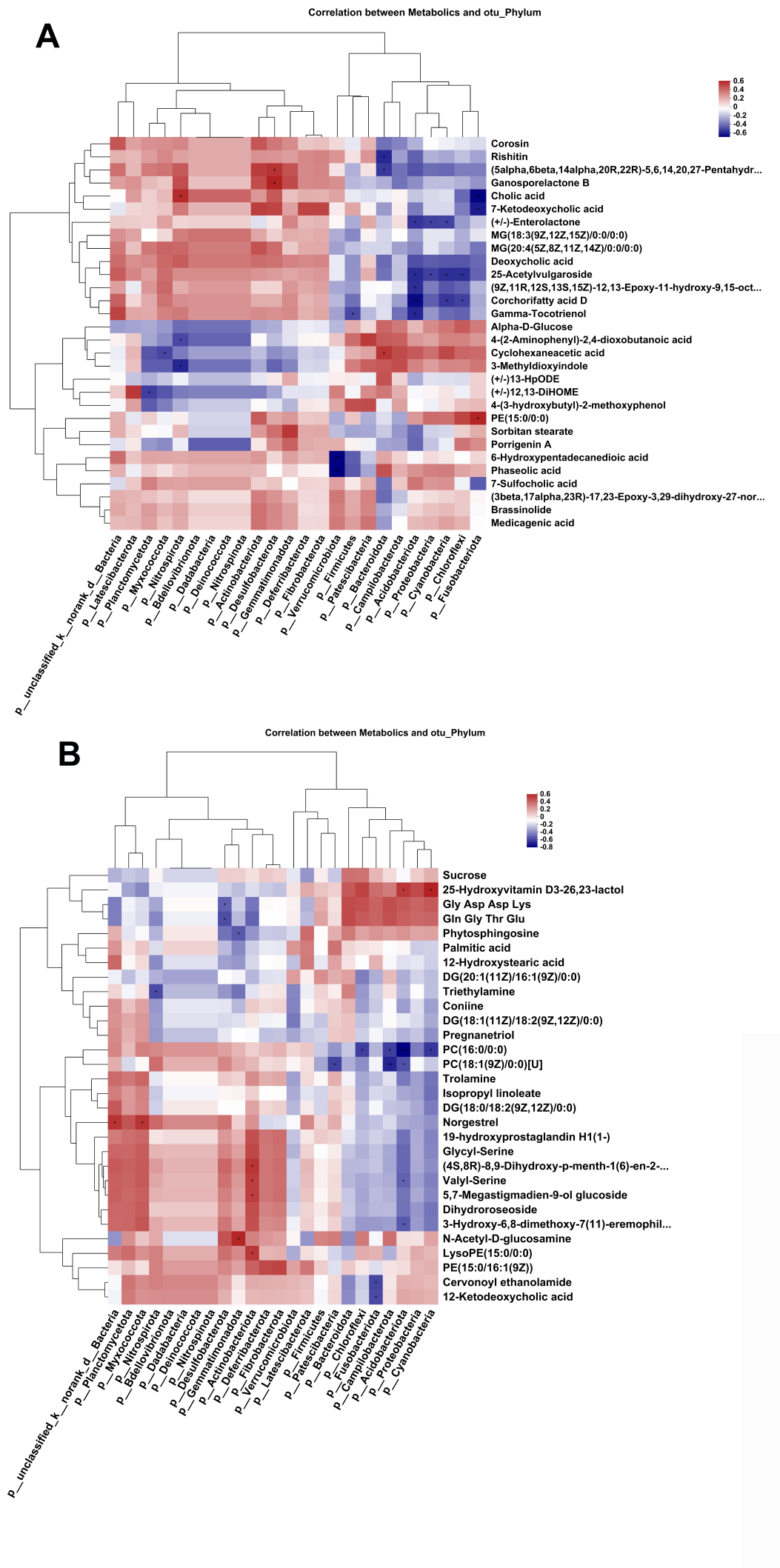


**Supplemental Figure S3.** The correlation analysis of UPLC-MS/MS metabolites with bacteria. (A) The correlation analysis of the metabolites under UPLC-MS/MS negative detection and bacteria on plylum level; (B) The correlation analysis of the metabolites UPLC-MS/MS positive detection on plylum level.

**
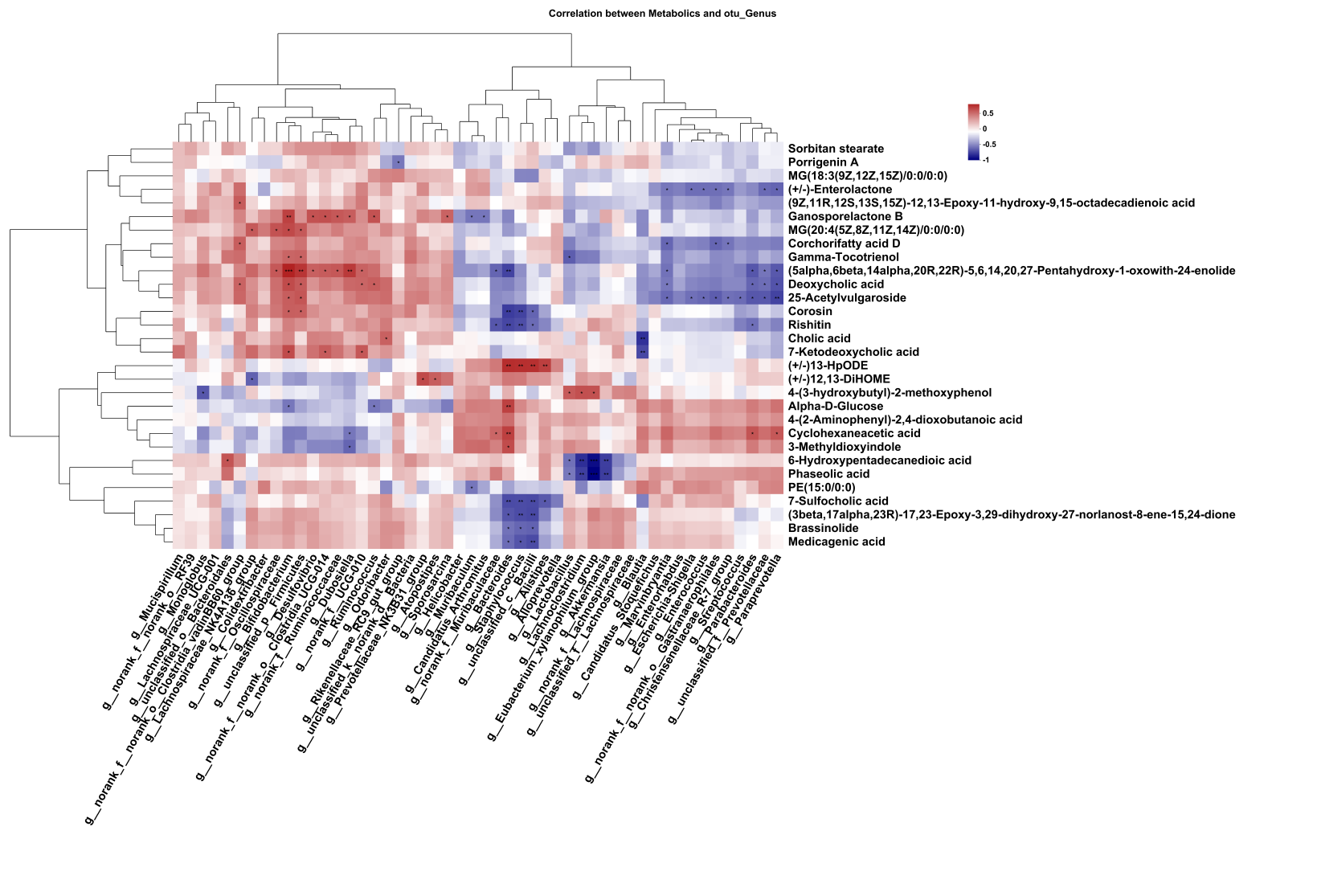
**

**Supplemental Figure S4.** The correlation analysis of metabolites under UPLC-MS/MS negative detection and intestinal bacteria on genus level.

**

**

**Supplemental Figure S5.** The correlation analysis of metabolites under UPLC-MS/MS positive detection and intestinal bacteria on genus level.


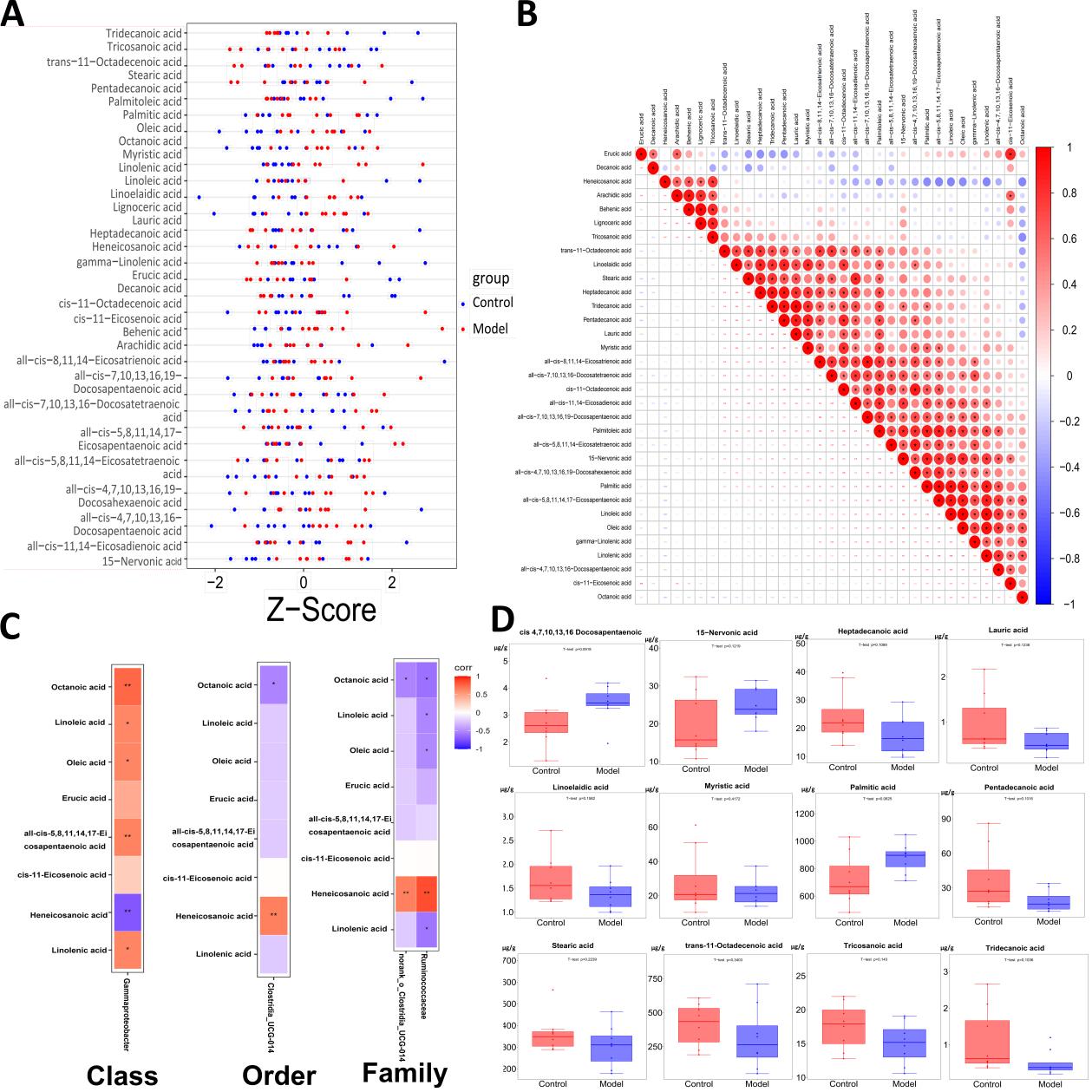


**Supplemental Figure S6.** The targeted metabolism. (A) Z-Core; (B) Differential metabolite association analysis; (C) Analysis of the association between differential bacteria and differential metabolites at the class, order and family levels; (D) The other targeted metabolites with differential levels.

**4. Materials and methods**

*4.9 16S rRNA sequencing of feces samples*

The concentration and purity were determined using NanoDrop2000 (Thermo Scientific, USA). The extracted DNA was used as the template, and PCR amplification of V3-V4 variable region of 16S rRNA gene using upstream primer 338F and downstream primer 806R carrying the Barcode sequence was performed [1]. The purified PCR products were constructed using the NEXTFLEX Rapid DNA-Seq Kit and sequenced by using Illumina's PE300/PE250 platform (Shanghai Meiji Biomedical Technology Co., Ltd.).

*4.10 Untargeted metabolism assay of feces samples in mice by GC-TOF/MS*

The supernatant was dried with nitrogen, and methoxypyridine hydrochloride solution (15 mg/mL) was added for the oximime reaction at 37°C in a shaking incubator for 90 min. Then, the BSTFA (containing 1% TMCS) derivatization reagent was added and reacted at 70°C for 60 min. The GC-MS analysis was performed by an Agilent 8890B gas chromatography coupled with an Agilent 5977B mass selective detector (Agilent, USA). 1 μL of sample was injected and separated by a DB-5MS capillary column (40 m × 0.25 mm × 0.25 μm, Agilent). The inlet temperature was 260°C with shunt ratio at 15: 1. The column temperature was programmed to hold at 60°C for 30 s and rise to 310°C at a rate of 8°C per minute. In mass detection, the ion source temperature was 230°C and the quadrupole temperature was 150°C. Full scan mode was used and the quality scanning was in the range of 50-500 (m/z).

*4.11 Untargeted metabolism assay of feces samples in mice by UPLC-MS/MS*

The samples were conducted on a Thermo UHPLC-Q Exactive HF-X system (Thermo Fisher Scientific, USA) equipped with an ACQUITY HSS T3 column (100 mm × 2.1 mm i.d., 1.8 μm, Waters, USA). The mobile phases was 0.1% formic acid in water: acetonitrile (95: 5, v/v) as the mobile phase A, and 0.1% formic acid in acetonitrile: isopropanol: water (47.5: 47.5: 5, v/v/v) as the mobile phase B. In positive detection mode, mobile phase B was increased from 0% to 20% (0-3 min), from 20% to 35% (3-4.5 min), from 35% to 100% (4.5-5 min), and maintained at 100% (5-6.3 min). In negative detection mode, mobile phase B was increased from 0% to 5% (0-1.5 min), from 5% to 10% (1.5-2 min), from 10% to 30% (2-4.5 min), from 30% to 100% (4.5-5 min), and the remaining gradients were the same as those in positive detection mode. The flow rate was 0.40 mL/min and the column temperature was 40°C. The quality detection was in the range of 70-1050 (m/z). The source temperature was 425°C, and the spray voltage was ±3500 V in positive and negative conditions.

*4.12 Targeted metabolomics profiling of feces samples by GC-TOF/MS assay*

The samples were extracted with isopropanol: n-hexane (2: 3, v/v containing 0.2 mg/L internal standard), homogenized at 40 Hz for 4 min, sonicated in ice water for 5 min, and centrifuged at 12,000 rpm/4°C for 15 min. The supernatant was produced and dried by nitrogen. The solution of methanol: trimethylsilyl diazomethane (1: 2, v/v) was added and the sample was dried, which was then re-dissolved by n-hexane, and centrifuged at 12,000 rpm for 1 min. At last, the supernatant was subjected to GC-MS analysis on 7890B GC System (Agilent, USA) and 5977B Mass Spectrometer (Agilent, USA), and separated on a DB-FastFAME capillary column (90 m × 250 μm × 0.25 μm). Helium was used as the carrier gas. The initial temperature was maintained at 50°C for 1 min, rise to 200°C and hold for 15 min. Then, the temperature was amped up to 210°C, hold for 1 min, rise to 230°C and hold for 15 min. The energy in electron impact mode was -70 eV, and mass spectrometry data were acquired under SIM mode in the range of 33-400 (m/z).

**References**

[1] Tan, J.; Gong, J.; Liu, F.; Li, B.; Li, Z.; You, J.; He, J.; Wu, S. Evaluation of an Antibiotic Cocktail for Fecal Microbiota Transplantation in Mouse. *Front. Nutr*. **2022**, 9, 918098.
